# Systematic Placement of the Enigmatic Southeast Asian Genus *Paralamium* and an Updated Phylogeny of Tribe Pogostemoneae (Lamiaceae Subfamily Lamioideae)

**DOI:** 10.3389/fpls.2021.646133

**Published:** 2021-04-16

**Authors:** Fei Zhao, Yi-Wen Wu, Bryan T. Drew, Gang Yao, Ya-Ping Chen, Jie Cai, En-De Liu, Bo Li, Chun-Lei Xiang

**Affiliations:** ^1^CAS Key Laboratory for Plant Diversity and Biogeography of East Asia, Kunming Institute of Botany, Chinese Academy of Sciences, Kunming, China; ^2^College of Life Sciences, Shaanxi Normal University, Xi’an, China; ^3^Department of Biology, University of Nebraska at Kearney, Kearney, NE, United States; ^4^South China Limestone Plants Center, College of Forestry and Landscape Architecture, South China Agricultural University, Guangzhou, China; ^5^Germplasm Bank of Wild Species in Southwest China, Kunming Institute of Botany, Chinese Academy of Sciences, Kunming, China; ^6^Research Centre of Ecological Sciences, College of Agronomy, Jiangxi Agricultural University, Nanchang, China

**Keywords:** Lamioideae, molecular phylogenetics, nutlet morphology, plastome phylogenomics, *Paralamium*, Pogostemoneae

## Abstract

*Paralamium* (Lamiaceae) is a monotypic genus within the subfamily Lamioideae and has a sporadic distribution in subtropical mountains of southeast Asia. Although recent studies have greatly improved our understanding of generic relationships within Lamioideae, the second most species-rich subfamily of Lamiaceae, the systematic position of *Paralamium* within the subfamily remains unclear. In this study, we investigate the phylogenetic placement of the genus using three datasets: (1) a 69,276 bp plastome alignment of Lamiaceae; (2) a five chloroplast DNA region dataset of tribe Pogostemoneae, and (3) a nuclear ribosomal internal transcribed spacer region dataset of Pogostemoneae. These analyses demonstrate that *Paralamium* is a member of Pogostemoneae and sister to the monotypic genus *Craniotome*. In addition, generic-level phylogenetic relationships within Pogostemoneae are also discussed, and a dichotomous key for genera within Pogostemoneae is provided.

## Introduction

Lamiaceae, as currently defined, contains about 7000 species and is subdivided into 12 subfamilies (Li et al., 2016; Li and Olmstead, 2017; Zhao et al., 2021). Lamioideae, containing at least 1260 species and about 61 genera, is the second-largest subfamily (after Nepetoideae) within Lamiaceae in terms of both the number of species and genera (Harley et al., 2004). Although the subfamily has a subcosmopolitan distribution, it is most common in southwest Asia and the Mediterranean region, China, and sub-Saharan Africa. During the past two decades, relationships and circumscription of constituent genera of Lamioideae have largely been clarified through both morphological (Abu-Asab and Cantino, 1992, 1994; Cantino, 1992a, b; Cantino et al., 1992; Ryding, 1994a, b, c, 1995, 1998, 2003, 2008; Salmaki et al., 2008; Xiang et al., 2013a; Seyedi and Salmaki, 2015) and molecular phylogenetic studies at various taxonomic levels (Wink and Kaufmann, 1996; Lindqvist and Albert, 2002; Scheen and Albert, 2007, Scheen et al., 2008, 2010; Bendiksby et al., 2011, 2014; Salmaki et al., 2012, 2013; Xiang et al., 2013b; Chen et al., 2014; Roy and Lindqvist, 2015; Li et al., 2016; Yao et al., 2016; Siadati et al., 2018). In particular, the molecular phylogenetics analyses of Scheen et al. (2010), Bendiksby et al. (2011), and Zhao et al. (2021) have dramatically improved our understanding of both tribal classification and character evolution within Lamioideae. Systematic positions of several enigmatic genera which were previously unplaced within Lamioideae have been recently elucidated (Scheen et al., 2010; Bendiksby et al., 2011; Chen et al., 2014; Roy and Lindqvist, 2015; Olmstead, 2016; Zhao et al., 2021), while a few genera, namely the rare and monotypic *Paralamium* Dunn. and *Metastachydium* Airy Shaw ex C.Y. Wu & H.W. Li, and *Roylea* Wall. ex Benth., remain unclassified at the tribal level because of insufficient molecular data available to date. The aforementioned *Paralamium* and *Metastachydium* have not been included in any published molecular phylogenetic study.

**FIGURE 1:**
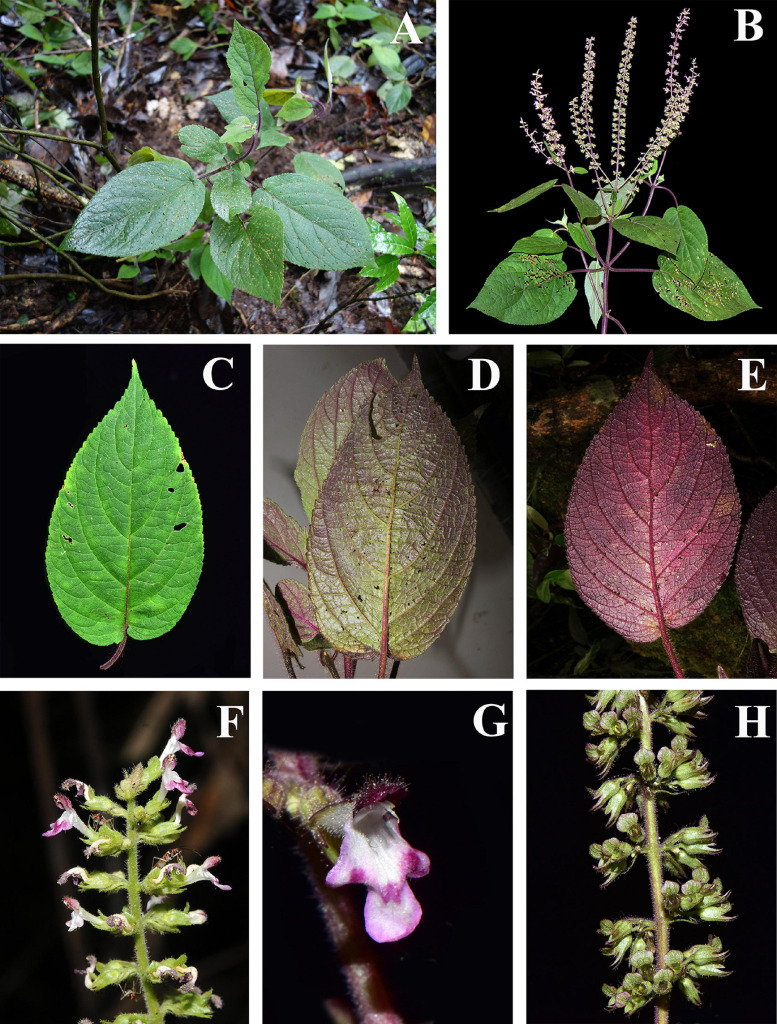
Morphology of *Paralamium griffithii*. **(A)** Habitat; **(B)** plant and inflorescence; **(C–E)** Leaf morphology, showing the color variation of leaf blades; **(F)** Lateral review of inflorescence; **(G)** Frontal view of flower; **(H)** Lateral of calyx. (Photo by XX Zhu).

The genus *Paralamium* was originally described by Dunn (1913) and reported to be endemic to southeast Asia with a sporadic distribution in humid regions of southwestern China (subtropical Yunnan), northern Vietnam, northern Burma, and eastern India (Assam) (Li and Hedge, 1994; Harley et al., 2004; Suddee and Paton, 2004). The genus is distinguished from other Lamioideae genera mostly based on calyx morphology. *Paralamium* has unequal calyx-lobes, with the posterior calyx tooth being the largest and having a truncate apex flanked by smaller triangular lateral lobes, and lanceolate-triangular anterior lobes (Figure 1). Harley et al. (2004) called this unique calyx morphology a 1/2/2 split, while Li and Hedge (1994) recognized this shape as a 1/4 split. In addition, this genus is characterized by possessing very small pollen grains with the polar length and/or equatorial width less than < 18 μm (Harley et al., 2004), which is an uncommon feature within Lamiaceae.

*Paralamium* is monotypic, with the sole species, *P. gracile* Dunn (1913) described on the basis of a specimen collected from Yunnan, China (*Henry 10636*). However, before the description of this species, Hooker (1885) described *Plectranthus griffithii* Hook.f. based on a collection from Eastern Assam, India (*Herb Griffith 4056*). After careful examination of the type materials, Suddee and Paton (2004) suggested that *Plectranthus griffithii* Hook.f. and *Paralamium gracile* Dunn. were conspecific. Thus, they formerly transferred the former species to *Paralamium* and a new combination, *Paralamium griffithii* (Hook.f.) S. Suddee & A.J. Paton, was created, making the latter species (*Paralamium gracile*) as a synonym.

The systematic position of *Paralamium* has been enigmatic ever since its original description. When establishing the genus, Dunn (1913) noted that the calyx is the “most striking” character of *Paralamium* and similar to *Orthosiphon* Benth. (Nepetoideae), *Coleus* Lour. (Nepetoideae) and *Teucrium* L. (Ajugoideae) by virtue of the following calyx characters: a broad upper calyx tooth with recurved decurrent margins and a conspicuously veined calyx tube. However, in the protologue for *Paralamium* (Dunn, 1913), the genus was also considered to be closely related to *Lamium* L. (Lamioideae) based on nutlet and corolla characters, hence the name “*Paralamium*” which can be translated to mean “resembling *Lamium.*” Studies on the genus after its original description have been scarce. Li (1977) placed *Paralamium* within subtribe Lamiinae of tribe Lamieae in subfamily Lamioideae sensu Briquet (1895–1897) based on its morphology provided in the protologue (Dunn, 1913). Later, Cantino and Sanders (1986) considered *Paralamium* as an anomalous genus within Lamiaceae because of its morphology similar to various genera in different subfamilies, but discreetly suggested that it could probably be related to *Lamium* based on their similar tricolpate and two-celled pollens observed by Abu-Asab and Cantino (1994). Harley et al. (2004) also placed *Paralamium* within Lamioideae in their comprehensive classification of Lamiaceae. In the most recent classifications of Lamioideae based on molecular data (Scheen et al., 2010; Bendiksby et al., 2011), *Paralamium* was provisionally treated as *incertae sedis* within Lamioideae but additionally suggested to be a member of tribe Pogostemoneae based on nutlet morphology (e.g., small glossy nutlets) (Bendiksby et al., 2011). While in the updated online synoptical classification of Lamiales, Olmstead (2016) placed *Paralamium* within tribe Stachydeae of Lamioideae. However, *Paralamium* has never been included in a published molecular phylogenetic analysis, making the above empirical placement of *Paralamium* within Lamioideae untested.

The main reason that *Paralamium* has not be included in any molecular phylogenetic studies is a lack of suitable leaf tissue for DNA extraction. However, during collecting expeditions in the Yunnan province of China in 2018 and 2019, we discovered two populations of *P. griffithii*. These collections allowed us to investigate the phylogenetic position of this monotypic and enigmatic genus based on molecular data. Here, using both plastid and nuclear ribosomal DNA markers, we present molecular phylogenetic analyses using different sampling strategies to finally establish the tribal affinities of *Paralamium* within Lamioideae and provide an updated phylogeny of the tribe Pogostemoneae. Furthermore, we provide a dichotomous key for genera within Pogostemoneae.

## Materials and Methods

### Field Collections

Specimens from two populations of *Paralamium griffithii* were collected from Malipo County (*Liu et al. 7859*) and Jinping County (*Z.Y. Cai and X.E. Ye czy-36*) within the Yunnan Province of China. Fresh leaves were collected and dried with silica gel. Voucher specimens were deposited in the Herbarium of Kunming Institute of Botany (KUN), Chinese Academy of Sciences.

### Taxon Sampling and Genetic Markers Selected

In order to better evaluate the systematic position of *Paralamium* and assess the phylogenetic relationships of this enigmatic genus and related genera, we experimented with three datasets. The first dataset included 79 plastid protein-coding genes within Lamiaceae (dataset CP79) aiming to confirm the subfamilial position of *Paralamium*. In total, 84 accessions from 84 species and 63 genera of Lamiaceae were included for this initial analysis, covering 11 of the 12 subfamilies recognized by Li et al. (2016) and Li and Olmstead (2017). The plastome of *P. griffithii* (*Z.Y. Cai and X.E. Ye czy-36*) was newly sequenced for this dataset. Outgroups of the dataset CP79 were selected from Mazaceae [*Mazus pumilus* (Burm. f.) Steenis)], Wightiaceae (*Wightia speciosissima* (D. Don) Merr.), Phrymaceae (*Phryma leptostachya* L. subsp. *asiatica* H. Hara), Paulowniaceae (*Paulownia coreana* Uyeki), and Orobanchaceae (*Castilleja paramensis* F. González et Pabón-Mora), according to recent Lamiales-wide phylogenies (Refulio-Rodriguez and Olmstead, 2014; Liu et al., 2020). GenBank accession numbers and the source publications for taxa in this dataset are provided in Supplementary Table 1. We used the phylogenetic results from this first set of analyses as a basis for a more focused second round of analyses.

Because the first set of analyses demonstrated that *Paralamium* has affinities with tribe Pogostemoneae of Lamioideae, we expanded the sampling of Pogostemoneae in a second round of analyses. These analyses focused on further exploring the placement of *Paralamium* within Pogostemoneae and explicating relationships among genera of the tribe. Chen et al. (2014) demonstrated that the monotypic genus *Holocheila* (Kudô) S. Chow is a member of Pogostemoneae, so we also included this genus for analysis. In total, for the first time, all 12 genera (including *Paralamium*) of Pogostemoneae were included as part of our Pogostemoneae-wide analyses. This comprehensive generic sampling offers the opportunity to clarify generic relationships of Pogostemoneae using five plastid regions (*mat*K, *rbc*L, *rps*16, *trn*H-*psb*A, *trn*L-*trn*F; dataset CP5) and the nuclear ribosomal internal transcribed spacer (dataset nrITS). In total, 56 sequences were newly sequenced for 13 species in 8 genera, while others were taken from previous studies (Chen et al., 2014; Yao et al., 2016) or downloaded from GenBank (Table 1). Outgroups for the dataset CP5 and the dataset nrITS were sampled from tribe Gomphostemmateae (*Chelonopsis souliei* (Bonati) Merr., *Gomphostemma lucidum* Wall. ex Benth., and *Gomphostemma* sp.) according to Yao et al. (2016).

**TABLE 1:** The voucher information of the taxa from Tribe Pogostemoneae analysis in this study, the GenBank accession numbers of the new sequenced are shown in the bold, other sequence were from the previous studies (NA = not available).

Taxa	Voucher information	nrITS	*mat*K	*rbc*L	*rps*16	*trn*H-*psb*A	*trn*L-trnF
*Chelonopsis souliei* (Bonati) Merr.	Xiang et al. 1638 (KUN)	**MW203029**	MT473743	MT473743	MT473743	MT473743	MT473743
*Gomphostemma lucidum* Wall. ex Benth.	Xiang et al. s.n. (KUN)	**MW203030**	MT473764	MT473764	MT473764	MT473764	MT473764
*Gomphostemma* sp.	G. Yao 298 (IBSC)	KR608723	KR608422	KR608487	KR608611	KR608546	KR608674
*Colebrookea oppositifolia* Sm. 1	G. Yao 342 (IBSC)	KR608732	KR608414	KR608479	KR608603	KR608538	KR608666
*Colebrookea oppositifolia* Sm. 2	G. Yao 367 (IBSC)	KR608733	KR608415	KR608480	KR608604	KR608539	KR608667
*Colebrookea oppositifolia* Sm. 3	G. Yao 385 (IBSC)	KR608734	KR608416	KR608481	KR608605	KR608540	KR608668
*Paralamium griffithii* (Hook.f.) Suddee & A.J. Paton 1	Liu et al. 7859 (KUN)	**MW203039**	**MW219635**	**MW219647**	**MW239150**	**MW239137**	**MW219659**
*Paralamium griffithii* (Hook.f.) Suddee & A.J. Paton 2	Z.Y. Cai and X.E. Ye czy-36 (IBSC)	**MW362555**	**MW201575**	**MW201575**	**MW201575**	**MW201575**	**MW201575**
*Craniotome furcata* (Link) Kuntze 1	G. Yao 346 (IBSC)	KR608730	KR608412	KR608477	KR608601	KR608536	KR608664
*Craniotome furcata* (Link) Kuntze 2	G. Yao 361 (IBSC)	KR608731	KR608413	KR608478	KR608602	KR608537	KR608665
*Holocheila longipedunculata* S. Chow 1	Xiang et al. 142 (KUN)	**MW203032**	AF315304	KF509868	KF509873	**MW239129**	KF509874 KF509880
*Holocheila longipedunculata* S. Chow 2	Peng et al. PLJ0048 (KUN)	**MW203033**	**MW219628**	**MW219640**	**MW239143**	**MW239130**	**MW219652**
*Achyrospermum wallichianum* (Benth.) Benth. ex Hook.f.	Liu et al. 16cs11840 (KUN)	**MW203028**	**MW219626**	**MW219638**	**MW239140**	**MW239127**	**MW219650**
*Eurysolen gracilis* Prain 1	G. Yao 366 (IBSC)	KR608735	KR608417	KR608482	KR608606	KR608541	KR608669
*Eurysolen gracilis* Prain 2	G. Yao 366 (IBSC)	KR608736	KR608418	KR608483	KR608607	KR608542	KR608670
*Leucosceptrum canum* Sm. 1	G. Yao 349 (IBSC)	KR608738	KR608419	KR608484	KR608608	KR608543	KR608671
*Leucosceptrum canum* Sm. 2	Peng et al. PLJ0049 (KUN)	**MW203034**	**MW219629**	**MW219641**	**MW239144**	**MW239131**	**MW219653**
*Comanthosphace ningpoensis* (Hemsl.) Hand.-Mazz.	Dong et al. HGNU-0864	**MW203030**	**MW219627**	**MW219639**	**MW239141**	**MW239128**	**MW219651**
*Comanthosphace japonica* (Miq.) S. Moore	NA	AB894375	HQ911407	NA	FJ854031	NA	FJ854274 FJ854161
*Rostrinucula dependens* (Rehder) Kudô	W. Fang fw11123 (KUN)	**MW203040**	**MW219636**	**MW219648**	**MW239151**	**MW239138**	**MW219660**
*Rostrinucula sinensis* (Hemsl.) C.Y.Wu	C.L. Xiang 355 (KUN)	**MW203041**	**MW219637**	**MW219649**	**MW239152**	**MW239139**	**MW219661**
*Microtoena* sp.	G. Yao 377 (IBSC)	KR608729	KR608410	KR608475	KR608599	KR608534	KR608662
*Microtoena urticifolia* Hemsl.	Y.P. Chen and Q.R. Zhao EM065 (KUN)	**MW203038**	**MW219634**	**MW219646**	**MW239149**	**MW239136**	**MW219658**
*Microtoena muliensis* C.Y.Wu	F. Zhao et al. LGH111 (KUN)	NA	**MW219632**	**MW219644**	**MW239147**	**MW239134**	**MW219656**
*Microtoena delavayi* Prain	Y.P. Chen EM599 (KUN)	**MW203035**	**MW219630**	**MW219642**	**MW239145**	**MW239132**	**MW219654**
*Microtoena moupinensis* (Franch.) Prain	Y.P. Chen EM631 (KUN)	**MW203036**	**MW219631**	**MW219643**	**MW239146**	**MW239133**	**MW219655**
*Microtoena robusta* Hemsl.	Y.P. Chen EM605 (KUN)	**MW203037**	**MW219633**	**MW219645**	**MW239148**	**MW239135**	**MW219657**
*Anisomeles indica* (L.) Kuntze 1	G. Yao 442 (IBSC)	KR608727	KR608408	KR608473	KR608597	KR608532	KR608660
*Anisomeles indica* (L.) Kuntze 2	G. Yao 448 (IBSC)	KR608728	KR608409	KR608474	KR608598	KR608533	KR608661
*Pogostemon barbatus* Bhoti & Ingr. 1	G. Yao 274 (IBSC)	KR608762	KR608452	KR608514	KR608639	KR608576	KR608701
*Pogostemon barbatus* Bhoti & Ingr. 2	G. Yao 446 (IBSC)	KR608763	KR608453	KR608515	KR608640	KR608577	KR608702
*Pogostemon auricularius* (L.) Hassk.	G. Yao 362 (IBSC)	KR608761	KR608451	KR608513	KR608638	KR608575	KR608700
*Pogostemon hispidocalyx* C.Y. Wu & Y.C.Huang	Expedition to QTP 9446 (KUN)	KR608780	KR608457	NA	KR608644	KR608581	KR608706
*Pogostemon litigiosus* Doan ex Suddee & A.J. Paton 1	V. D. Nong 31712077 (IBSC)	KR608776	KR608458	KR608519	KR608645	KR608582	KR608707
*Pogostemon litigiosus* Doan ex Suddee & A.J. Paton 2	V. D. Nong 6467 (IBSC)	KR608777	KR608459	KR608520	KR608646	KR608583	KR608708
*Pogostemon brachystachyus* Benth. 1	G. Yao 358 (IBSC)	KR608775	KR608455	KR608517	KR608642	KR608579	KR608704
*Pogostemon brachystachyus* Benth. 2	G. Yao 359 (IBSC)	KR608774	KR608454	KR608516	KR608641	KR608578	KR608703
*Pogostemon fraternus* Miq.	Syn. 7655 (KUN)	KR608781	KR608461	NA	KR608648	KR608585	KR608710
*Pogostemon rogersii* N.E.Br.	Phillips 3855 (K)	KR608782	KR608460	NA	KR608647	KR608584	KR608709
*Pogostemon quadrifolius* (Roxb. ex D.Don) F.Muell.	F. G. Dickason 8194 (A)	KR608773	KR608456	KR608518	KR608643	KR608580	KR608705
*Pogostemon aquaticus* (C.H.Wright) Press	Bidgood et al. 3387 (K)	KR608767	KR608468	KR608527	KR608655	KR608592	KR608717
*Pogostemon yatabeanus* (Makino) Press	G. Yao 285 (IBSC)	KR608766	KR608467	KR608526	KR608654	KR608591	KR608716
*Pogostemon linearis* (Benth.) Kuntze 1	G. Yao 348 (IBSC)	KR608764	KR608462	KR608521	KR608649	KR608586	KR608711
*Pogostemon linearis* (Benth.) Kuntze 2	G. Yao 348 (IBSC)	KR608765	KR608463	KR608522	KR608650	KR608587	KR608712
*Pogostemon cruciatus* (Benth.) Kuntze	T. P. Zhu 528 (KUN)	KR608771	KR608466	KR608525	KR608653	KR608590	KR608715
*Pogostemon petelotii* Doan ex Gang Yao, Y.F. Deng & X.J.Ge	T. Sorensen et al. 6313 (KUN)	KR608772	KR608470	KR608529	KR608657	KR608594	KR608719
*Pogostemon stellatus* (Lour.) Kuntze	B. Z. Xiao 4826 (K)	KR608768	KR608464	KR608523	KR608651	KR608588	KR608713
*Pogostemon crassicaulis* (Benth.) Press	J. T. Yin 594 (HITBC)	KR608770	KR608469	KR608528	KR608656	KR608593	KR608718
*Pogostemon sampsonii* (Hance) Press	G. Yao 273 (IBSC)	KR608769	KR608465	KR608524	KR608652	KR608589	KR608714
*Pogostemon heyneanus* Benth.	G. Yao 297 (IBSC)	KR608751	KR608427	KR608492	KR608616	KR608551	KR608679
*Pogostemon cablin* (Blanco) Benth. 1	G. Yao 292 (IBSC)	KR608752	KR608439	KR608504	KR608628	KR608563	KR608691
*Pogostemon cablin* (Blanco) Benth. 2	G. Yao 296 (IBSC)	KR608756	KR608443	KR608508	KR608632	KR608567	KR608695
*Pogostemon parviflorus* Benth. 1	G. Yao 365 (IBSC)	KR608749	KR608436	KR608501	KR608625	KR608560	KR608688
*Pogostemon parviflorus* Benth. 2	G. Yao 365 (IBSC)	KR608750	KR608437	KR608502	KR608626	KR608561	KR608689
*Pogostemon plectranthoides* Desf.	W. Koelz 4153 (US)	KR608760	KR608446	KR608509	KR608634	KR608570	KR608696
*Pogostemon plectranthoides* Desf.	G. Yao 449 (IBSC)	KR608758	KR608447	KR608510	KR608635	KR608571	KR608697
*Pogostemon xanthiiphyllus* C. Y. Wu et Y. C. Huang	H. T. Tsai 59-10586 (KUN)	KR608746	KR608428	KR608493	KR608617	KR608552	KR608680
*Pogostemon formosanus* Oliv. 1	C. H. Lin 370 (US)	KR608744	KR608434	KR608499	KR608623	KR608558	KR608686
*Pogostemon formosanus* Oliv. 2	R.Q. Gao and S.H. Lai 710 (PE)	KR608779	KR608435	KR608500	KR608624	KR608559	KR608687
*Pogostemon glaber* Benth. 1	G. Yao 364 (IBSC)	KR608739	KR608429	KR608494	KR608618	KR608553	KR608681
*Pogostemon glaber* Benth. 2	G. Yao 386 (IBSC)	KR608741	KR608430	KR608495	KR608619	KR608554	KR608682
*Pogostemon chinensis* C.Y. Wu & Y.C. Huang 1	J. Chen 656 (KUN)	KR608743	KR608426	KR608491	KR608615	KR608550	KR608678
*Pogostemon chinensis* C.Y. Wu & Y.C. Huang 2	G. Yao 445 (IBSC)	KR608742	KR608449	KR608512	KR608637	KR608573	KR608699
*Pogostemon septentrionalis* C.Y.Wu & Y.C.Huang 1	G. Yao 264 (IBSC)	KR608747	KR608432	KR608497	KR608621	KR608556	KR608684
*Pogostemon septentrionalis* C.Y.Wu & Y.C.Huang 2	G. Yao 272 (IBSC)	KR608748	KR608433	KR608498	KR608622	KR608557	KR608685
*Pogostemon amaranthoides* Benth.	J. Chen 668 (KUN)	KR608745	KR608425	KR608490	KR608614	KR608549	KR608677

### DNA Extraction, Amplification, and Sequencing

Total genomic DNA was extracted from fresh or silica-gel-dried leaf fragments using the CTAB procedure of Doyle and Doyle (1987), then dissolved in double-distilled water and kept at −20°C for future polymerase chain reaction (PCR) amplification.

Primers and PCR thermal cycler settings for *mat*K and *rbc*L followed Chen et al. (2014), and those for nrITS, *trn*L-*trn*F, *rps*16, and *trn*H-*psb*A were as described by Xiang et al. (2013b). Amplified PCR products were visualized on 1% TBE agarose gel, stained with ethidium bromide and then sequenced by an ABI-PRISM3730 sequencer after purification with a QIAquick PCR purification Kit (BioTek, Beijing, China). Voucher information for newly sequenced species and GenBank accession numbers for all sequences used in the current study are listed in the Table 1.

### Plastome Sequencing, Assembly, Annotation, and Gene Region Extraction

The DNA concentration of *Paralamium griffithii* was at least 35 ng/μL as measured by a NanoDrop spectrophotometer 2000 (Thermo Scientific, Carlsbad, CA, United States). DNA integrity was detected and purified by 1% Agarose Gel Electrophoresis for 40 min at 150 V. Subsequently, the DNA samples were sheared into 300 bp fragments for paired-end library construction according to manufacturer’s instructions (Illumina, San Diego, CA, United States), details are provided in Zhao et al. (2020a).

Prior to genome assembly, adapter sequences and low-quality reads were removed using the ea-utils package^1^. Quality control of raw sequence reads was carried out using FastQC 0.11.8 (Andrews, 2018) with the parameter set as Q ≥ 25. We used the GetOrganelle pipeline (Jin et al., 2020) for the *de novo* assembling. The software Bandage v. 0.8.1 (Wick et al., 2015) was employed for contig visualization and editing. Lastly, in order to validate the assembly error, the raw reads were mapped to the assembled plastid genome sequences by the Bowtie2 (Langmead and Salzberg, 2012) plugin in Geneious v. 11.0.3 (Kearse et al., 2012). In addition to the newly sequenced plastome of *Paralamium griffithii* and downloaded plastomes of 54 species from GenBank (Supplementary Table 1), 32 data from the Sequences Read Archive (SRA) were included for reassembling.

The Initial annotations were implemented in the Plastid Genome Annotator (PGA) (Qu et al., 2019), and the published plastome of *Phlomoides betonicoides* (Diels) Kamelin & Makhm (MN617020; Zhao et al., 2020b) was set as a reference, then Geneious v.11.0.3 (Kearse et al., 2012), and tRNAscan-SE service (Lowe and Chan, 2016) were used adjusting of the putative starts, stops, intron positions, and tRNA boundaries as described by Zhao et al. (2021) and Xiang et al. (2020). Finally, the circular physical map of the plastome of *Paralamium* (Supplementary Figure 1) was drawn by the Organellar Genome DRAW tool (Lohse et al., 2013). The coding regions (CR) were extracted from the annotated complete plastome sequences for phylogenetic analyses.

### Sequence Alignment and Phylogenetic Analyses

Sequences were assembled and edited using Geneious v.11.0.3 (Kearse et al., 2012). Sequences were aligned using MAFFT v.7.221 (Katoh and Standley, 2013), and then adjusted manually in PhyDE v.0.9971 (Müller et al., 2010) for minor corrections. All datasets were submitted to TreeBASE (study ID: S27475).

Since topological incongruence between the combined cpDNA and nrITS data was reported in Yao et al. (2016), the nrITS and cpDNA datasets were not combined for analyses here. However, because plastome regions typically have a shared genetic history, the five plastid DNA regions were combined for phylogenetic analyses. All datasets were analyzed using Maximum Likelihood (ML) and Bayesian Inference (BI) algorithms on the CIPRES Science Gateway (^2^ Miller et al., 2010). The ML analyses were implemented with RAxML v.8.2.9 (Stamatakis, 2014), bootstrap probabilities were generated by conducting 1000 bootstrap iterations, and details for parameter settings are described by Xiang et al. (2020). Bayesian inference analyses were performed using MrBayes v.3.2.2 (Ronquist et al., 2012). The best-fit nucleotide substitution models were selected under the Akaike Information Criterion (AIC) using jModelTest v.3.7 (Posada, 2008). The models used were the GTR+I+G for dataset CP79, TVM+I+G for dataset CP5, and for the nrITS dataset. In addition, a partitioned strategy for the dataset CP5 also used for Bayesian inference analyses (GTR + G for *mat*K, GTR + I for *rbc*L, TVM + G for *rps*16, TPM1uf + G for *trn*H-*psb*A, GTR + I for *trn*L*-trn*F). Specific steps for analyses are described in detail in Chen et al. (2016) and references provided therein. Finally, we used FigTree v.1.4.2 (Rambaut, 2014) to visualize and edit all resulting trees. We defined branches with posterior probabilities (PP) ≥ 0.95 and bootstrap values (BS) ≥ 80% as strongly supported, PP = 0.90–0.95 and BS = 70–80% as moderately supported, while PP < 0.90 and BS < 70% were defined as weakly supported.

### Nutlets Morphology

Mature nutlets were collected from both wild-collected or herbarium plant specimens from the Germplasm Bank of Wild Species in Southwest China, Kunming Institute of Botany, for light microscope (LM) and scanning electron microscope (SEM) observation. With the exception of the monotypic *Holocheila*, nutlet morphology was examined for all genera of Pogostemoneae. Based on previous studies (Yao et al., 2016; Wang, 2018) and our phylogenetic analyses, three species representing both major clades of *Pogostemon* were selected, and four species from *Microtoena* were sampled. For each species, at least five mature nutlet samples were examined. In total, nutlets of 17 species representing 11 out of 12 genera of Pogostemoneae were included for morphological investigation. Measurements and LM investigation were done with the Keyence VHX-6000 digital microscope. For SEM examination, mericarps were directly affixed to stubs with double-sided tape and the sputter-coated with gold-palladium. Observations were conducted using ZEISS EVO LS10 scanning electron microscope (Carl ZEISS NTS, Germany) with 10 kV voltage (Kunming Institute of Botany, Yunnan, China). Nutlets terminology followed Moon et al. (2009).

## Results

### Genome Assembly, Features and Gene Content of *Paralamium griffithii*

The newly sequenced and annotated plastome was submitted to the National Center for Biotechnology Information (NCBI) database with the accession number MW201575. Illumina paired-end sequencing generated 20,321,882 clean reads, with coverage of 179 × for *P. griffithii*. The plastome size was 152,664 bp and displayed the typical quadripartite structure consisting of a pair of IR regions (25,617 bp) separated by the large single copy (LSC; 83,788 bp) and small single copy (SSC; 17,642 bp) regions (Supplementary Figure 1). In total, 114 unique genes (80 protein-coding genes, 30 tRNAs, and 4 rRNAs; Supplementary Table 2) were identified (duplicated genes in IR regions were counted only once). We used 79 common protein-coding genes for phylogenetic analyses based on Zhao et al. (2021) with the exclusion of the *ycf*15 gene because it could not be extracted from most plastome reassembed from SRA database.

### Sequence Characterization

Properties for different datasets are summarized in Table 2. The aligned length of the combined 79 protein coding regions (CP79) was 70,100 bp. Removal of ambiguous sites and single taxon insertions resulted in an aligned length of 69,276 bp, of which 47,566 sites were constant (68.66%). The aligned regions and the excluded ambiguous sites of the individual loci are listed in Supplementary Table 3.

**TABLE 2:** The statistics of all datasets for phylogenetic analysis.

Datasets	No. Taxa	Nucleotides (with ambiguous sites excluded) [bp]	GC content (%)	No. constant sites [bp]	No. variable sites [bp]	No. parsimony- informative sites [bp]
CP79	85	69,276	38.30%	47566 (68.66%)	21,710 (31.34%)	13,285 (19.18%)
*mat*K	66	832	33.90%	683 (82.09%)	149 (17.91%)	113 (13.58%)
*psb*A*-trn*H	65	292	32.60%	202 (69.18%)	90 (30.82%)	71 (24.32%)
*rbc*L	65	574	44.10%	535 (93.21%)	39 (6.79%)	35 (6.1%)
*rps*16	66	861	34.70%	693 (80.49%)	168 (19.51%)	123 (14.29%)
*trn*L*-trn*F	66	880	35.90%	774 (87.96%)	106 (12.04%)	68 (7.73%)
CP5	66	3,439	36.20%	2,887 (83.95%)	552 (16.05%)	410 (11.92%)
nrITS	65	656	62.80%	305 (46.49%)	351 (53.51%)	279 (42.53%)

In the second set of analyses, the combined cpDNA dataset was 3,439 bp (832 bp for *mat*K, 574 bp for *rbc*L, 880 bp for *trn*L*-trn*F, 861 bp for *rps*16, and 292 bp for *trn*H*-psb*A) after excluding ambiguously aligned characters. The nrITS matrix contained 656 aligned positions (Table 2).

### Phylogenetic Analysis

For each combined dataset (CP79, CP5, and nrITS), ML and BI analyses yielded identical topologies, respectively (Figures 2–4; Supplementary Figures 2–8). Therefore, only the trees resulting from maximum likelihood analysis of each dataset are presented, with posterior probability values from BI analyses indicated.

**FIGURE 2:**
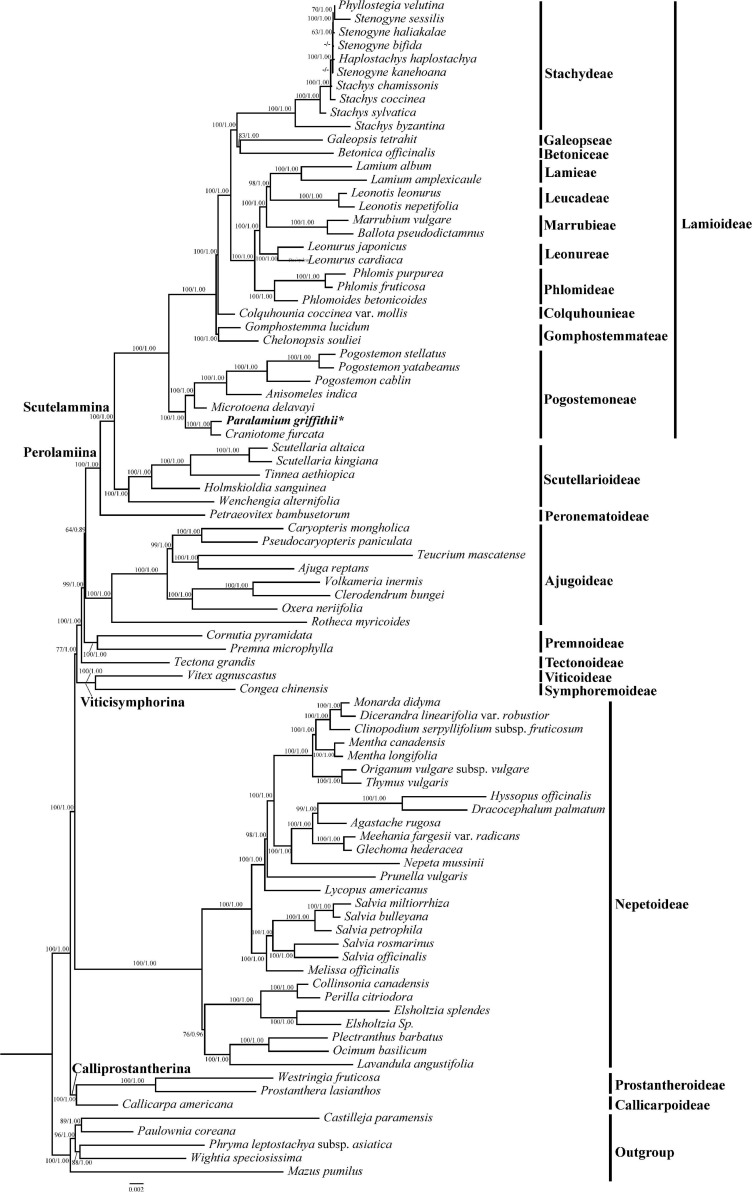
Phylogeny of Lamiaceae inferenced by maximum likelihood (ML) based on 79 coding regions (dataset CP79), with ambiguously aligned sites excluded from analysis. Bootstrap values ≥ 50% in ML and posterior probability values ≥ 0.90 in BI analyses displayed on the branch follow the order ML_*BS*_/BI_*PP*_ (“-” indicates a support value BS < 50% or PP values < 0.9). Subfamilial classification of Lamiaceae is based on Li et al. (2016) and Li and Olmstead (2017).

**FIGURE 3:**
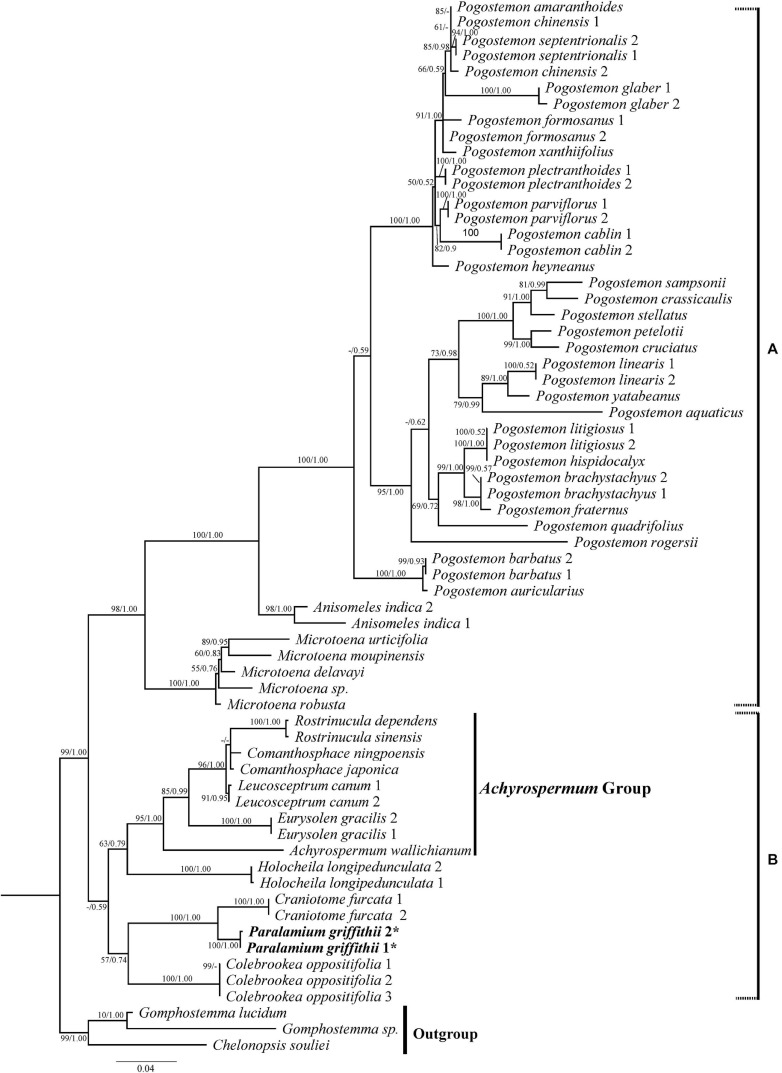
Phylogenetic relationships of Pogostemoneae based on the nrITS dataset. The support values (BS/PP) are indicated above branches. BS values < 50% and PP support < 90% indicated by -. The outgroup and other major groups are labeled at the right.

**FIGURE 4:**
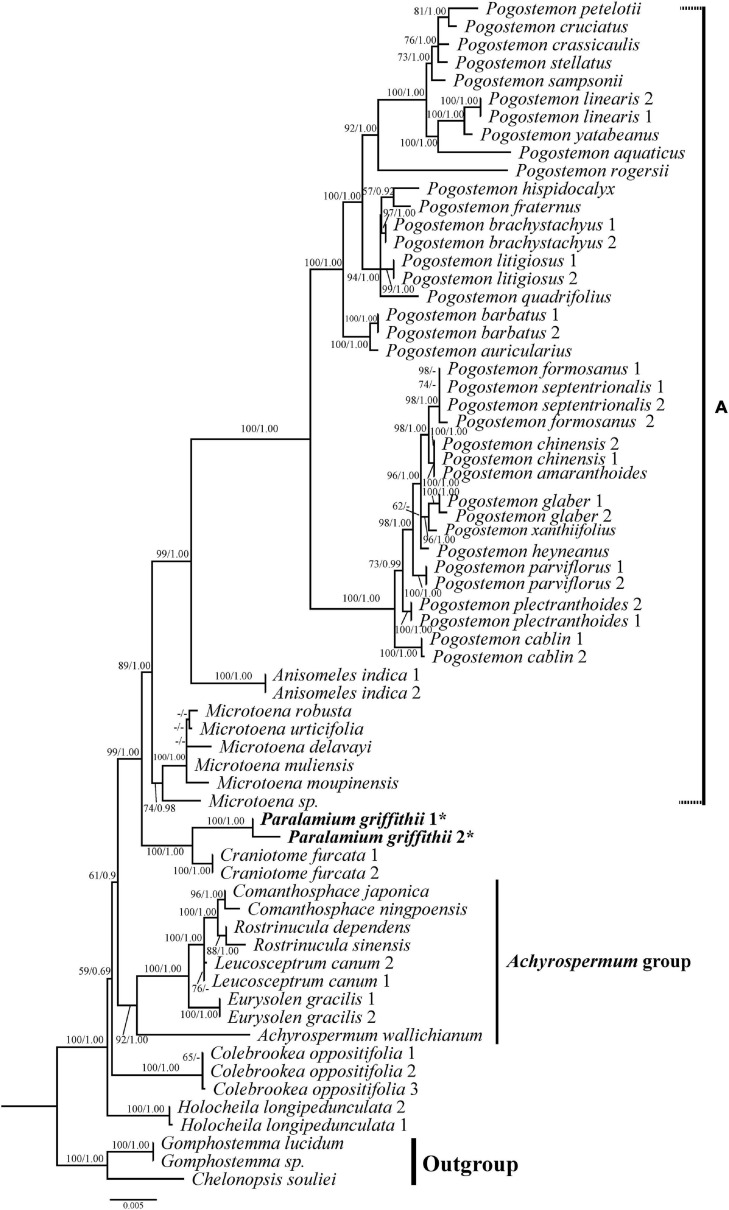
Phylogenetic relationships of Pogostemoneae based on the dataset CP5. The support values (BS/PP) are indicated above branches. BS values < 50% and PP support < 90% indicated by -. The outgroup and other major groups are labeled at the right.

In our phylogenetic analyses based on 79 coding plastome sequences (CP79), Lamiaceae are supported as monophyletic (Figure 2; ML-BS = 100%/BI-PP = 1.00; all support values follow this order hereafter) and subfamilial relationships are identical to those recovered by Zhao et al. (2021), and 11 tribes were recovered within Lamioideae (Figure 2). In all analyses, the focal species *Paralamium griffithii* was sister to *Craniotome furcata* (Link) Kuntze (100%, 1.00) within tribe Pogostemoneae of subfamily Lamioideae.

This recognition guided the second set of analyses, which aimed to further clarify the position of *Paralamium*, reassess generic relationships within Pogostemoneae, and update the phylogeny of Pogostemoneae by including as comprehensive taxon sampling as possible using both nrITS and cpDNA data. In all analyses, Pogostemoneae is robustly supported as monophyletic (Figures 3, 4), but the topologies differed between the nrITS and cpDNA phylogenetic trees. In the nrITS phylogeny, Pogostemoneae was found to have two major clades (labelled A and B in Figure 3). Clade A, or the *Pogostemon* group, includes *Pogostemon* Desf., *Anisomeles* R. Br., and *Microtoena* Prain, in which the former two genera formed a clade (100%, 1.00) sister to *Microtoena* (98%, 1.00). Clade B is poorly supported (59%, -) and includes nine genera. Clade B in turn is comprised of two subclades: one containing *Colebrookea* Sm., *Paralamium* + *Craniotome* Rchb., weakly supported (57%, -); and another subclade composed of *Holocheila* and the “*Achyrospermum* group” (i.e., *Achyrospermum* Blume, *Eurysolen* Prain, *Leucosceptrum* Sm., *Comanthosphace* S. Moore, and *Rostrinucula* Kudô), also poorly supported (63%, -).

All analyses based on the combined cpDNA dataset (CP5) also strongly supported the monophyly of Pogostemoneae (Figure 4; 100%, 1.00). At this point in the Pogostemoneae topology, the two samples of *Holocheila* formed a well-supported clade (100%, 1.00) and were recovered as sister to the remaining Pogostemoneae, which formed a weakly supported clade (59%, -). This “remaining Pogostemoneae” clade included *Colebrookea* (100%, 1.00), the *Achyrospermum* group (92%, 1.00), *Paralamium* + *Craniotome* (100%, 1.00), and Clade A (i.e., the *Pogostemon* group, 89%, 1.00).

### Nutlets Morphology

**FIGURE 5:**
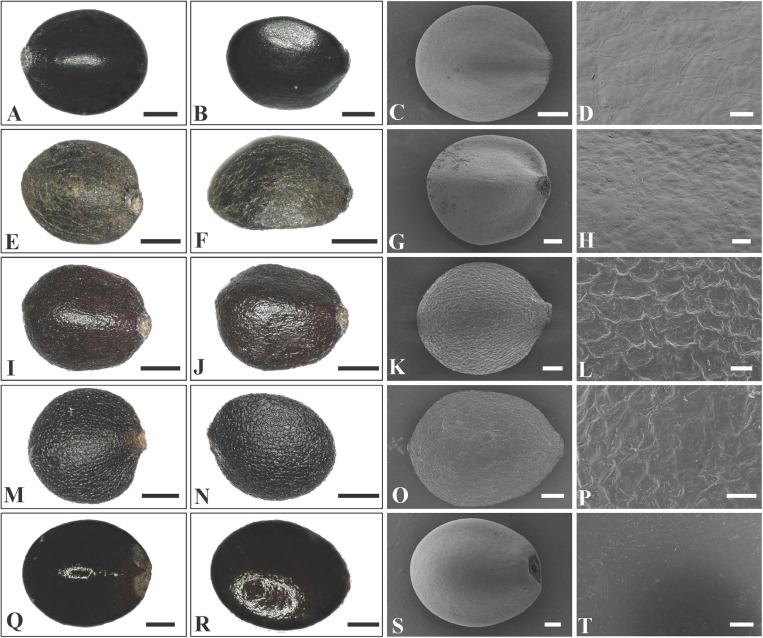
LM and SEM micrographs of mericarps of *Pogostemon* and *Anisomeles*. **(A–D)** *P. chinensis* (*Guo et al., 12CS5297*), **(E–H)** *P. glaber* (*Li Yongliang, LiYL1637*), **(I–L)** *P. brachystachyus* (*Sun Xingxu, SunXX154*), **(M–P)** *P. amaranthoides* (*Sun Xingxu, SunXX148*), **(Q–T)** *Anisomeles indica* (*Liu et al., SCSB-B-0000020*).—Scale bars: **(A–C,E–F,I–J,M–N,S)** 200 μm; **(D,H,L,P)** 20 μm; **(G,K,O)** 100 μm; **(Q,R)** 400 μm; **(T)** 40 μm.

**FIGURE 6:**
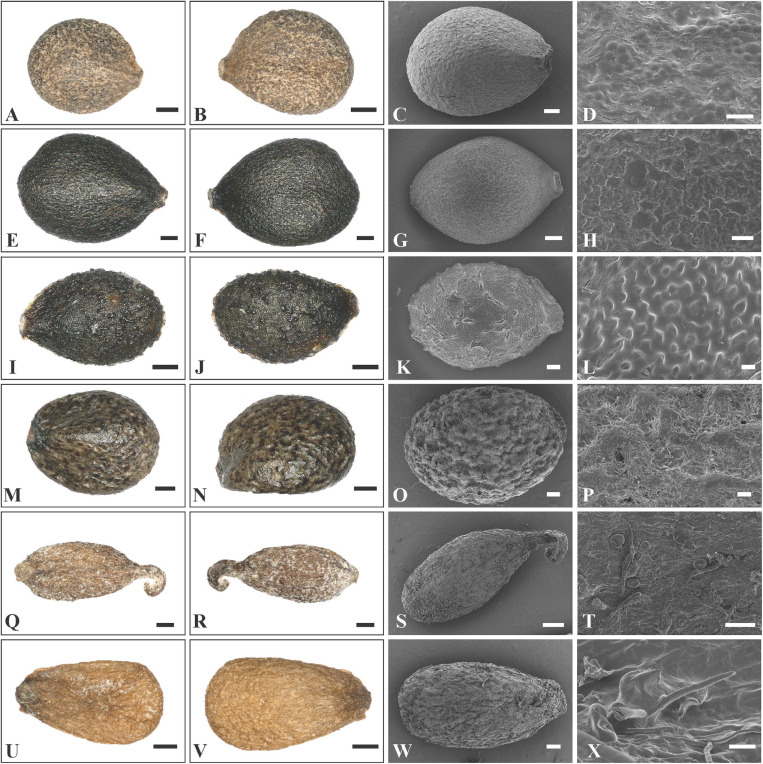
LM and SEM micrographs of mericarps of *Microtoena*, *Rostrinucula*, and *Comanthosphace*. **(A–D)** *M. delavayi* (*Wang et al., SCSB-TBG-147*), **(E–H)** *Microtoena praineana* (*Yi Sirong and Tan Qiuping, YISR432*), **(I–L)** *M. esquirolii* (*Cai Jie and Zhang Ting, 12CS5726*), **(M–P)** *M. stenocalyx* (*Li et al*., *LiYL1849*), **(Q–T)** *Rostrinucula sinensis (Zhang Daigui, 3756*), **(U–X)** *Comanthosphace ningpoensis* (*Zhu et al., ZhuXX121*). — Scale bars: **(A–B,Q–S,U–V)** 400 μm; **(C,E–F,I–J,M–N,T,W)** 200 μm; **(D)**: 40 μm, **(G,K,O)** 100 μm; **(H,L,P,X)** 20 μm.

The nutlets of the genera in clade A (Figure 3) are glossy and smooth (Figures 5, 6A–P) compared with those of genera in clade B (Figure 3). As reported previously (Ryding, 1994a; Bongcheewin et al., 2017), the nutlets of *Pogostemon* (Figures 5A–P) and *Anisomeles indica* (L.) Kuntze (Figures 5Q–T) are orbicular to subglobose, dark-brown to black, and the surface is very smooth (*P*. *chinensis* C.Y. Wu et Y.C. Huang, Figures 5A–D; *P. glaber* Benth., Figures 5E–H) or finely striato-reticulate (*P*. *brachystachyus* Benth., Figures 5I–L; *P*. *amaranthoides* Benth.; Figures 5M–P). In *Microtoena* (Figures 6A–P), nutlets are ovoid or subglobose, brown to black, glossy, and the surface is relatively smooth (*M. delavayi* Prain, Figures 6A–D; *M*. *prainiana* Diels, Figures 6E–H; *M*. *stenocalyx* C.Y. Wu et S.J. Hsuan, Figures 6M–P), or finely granulated (*M. esquirolii* H. Lév.; Figures 6I–L).

**FIGURE 7:**
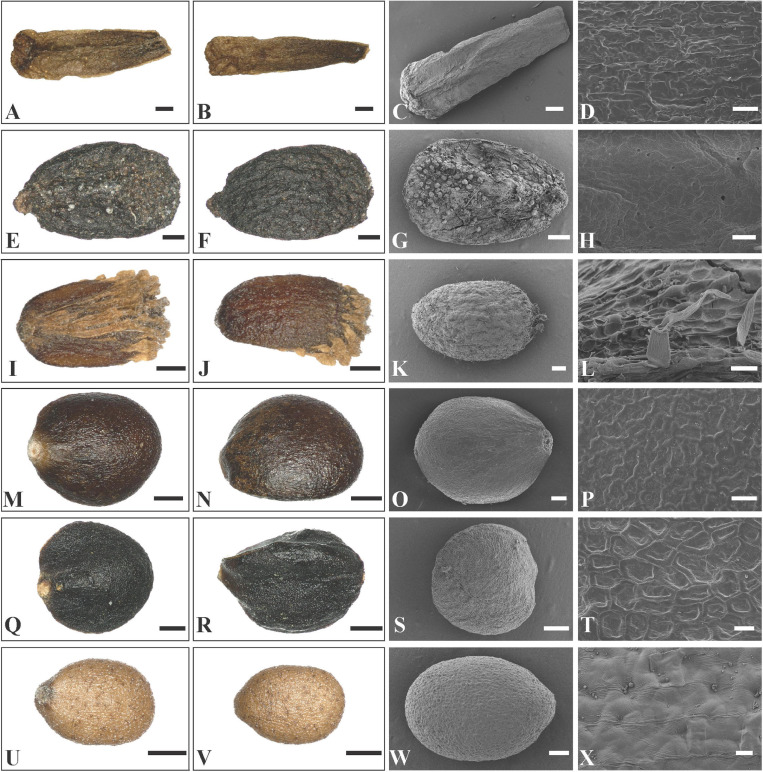
LM and SEM micrographs of mericarps of *Leucosceptrum, Eurysolen*, *Achyrospermum, Craniotome*, *Paralamium* and *Colebrookea.* **(A–D)** *Leucosceptrum canum* (*Li Yongliang, YDDXS00058*), **(E–H)** *Eurysolen gracilis (Chen Yaping and Jiang Lei, EM1436*), **(I–L)** *Achyrospermum wallichianum* (*Liu et al., 16CS11840*), **(M–P)** *Craniotome furcata* (*Liu et al., 16CS11942*), **(Q–T)** *Paralamium griffithii* (*Liu et al., 7859*), **(U–X)** *Colebrookea oppositifolia* (*Li Yongliang, YDDXS1001*). — Scale bars: **(A–C,I,J)** 400 μm; **(D,H,L,P)** 20 μm; **(E–G,K,M,N,Q,R,U,V)** 200 μm; **(O,S,W)** 100 μm; **(T,X)** 10 μm.

In *Rostrinucula*, the nutlets are narrowly ellipsoid with curved hook-like apices, brown, pubescent outside with glands and eglandular trichomes (*R. sinensis* (Hemsl.) C.Y. Wu, Figures 6Q–T). Nutlets of *Comanthosphace* are obovate, light brown, and the surface is rough and has subsessile and eglandular trichomes (*C. ningpoensis* (Hemsl.) Hand.-Mazz., Figures 6U–X). In *Leucosceptrum canum* Sm. the nutlets are oblong, brown, with sharp edges or ribs apically, and a surface more or less smooth but with sparse subsessile glands (Figures 7A–D). Nutlets of *Eurysolen* are also obovate, dark brown, dull, and densely glandular along the ventral side (Figures 7E–H). Only one species of *Achyrospermum, A. wallichianum* (Benth.) Benth. ex Hook. f., was included for this study. *Achyrospermum wallichianum* has somewhat elliptic light brown nutlets that are hairy at apex and reticulate on the surface (Figures 7I–L). Nutlets of *Craniotome* (Figures 7M–P) and *Paralamium* (Figures 7Q–T) are subspheric, brown and black respectively, and slightly reticulate outside. Nutlets of *Colebrookea* (Figures 7U–X) are obovoid to oblong, light brown, with apices and fruit navels densely covered with glands, and a surface that is smooth and sometimes with subsessile glands.

## Discussion

### *Paralamium* as a Member of Pogostemoneae in Subfamily Lamioideae

The resulting topologies of Lamiaceae from the dataset CP79 are consistent with that of previous studies (Li et al., 2016) based on five cpDNA regions and relationships among these subfamilies are well resolved. Moreover, all tribes of Lamioideae are strongly supported as monophyletic (Figure 2), which is in concordance with previous studies (Scheen et al., 2010; Bendiksby et al., 2011; Roy and Lindqvist, 2015; Zhao et al., 2021).

Cantino and Sanders (1986) considered *Paralamium* as an anomalous genus within Lamiaceae because of its morphological similarities to genera from various subfamilies (i.e. *Orthosiphon* and *Coleus* of Nepetoideae, *Ajuga* of Ajugoideae, and *Lamium* of Lamioideae). However, the presence of tricolpate and two-celled pollens in *Paralamium* suggested its placement within Lamioideae (Cantino and Sanders, 1986). The genus was suggested to be closely related to Pogostemoneae by Bendiksby et al. (2011) based on nutlet morphology, but they explicitly treated it as *incertae sedis* within Lamioideae due to the lack of molecular phylogenetic data. Here, both the plastid and nuclear DNA data (Figures 2–4) support that *Paralamium* is a member of tribe Pogostemoneae, and is sister to the monotypic genus *Craniotome*.

Previous studies based on cpDNA sequences showed that *Craniotome* grouped with *Microtoena*, *Anisomeles*, and *Pogostemon* (Scheen et al., 2010; Bendiksby et al., 2011; Chen et al., 2014). Using low-copy nuclear pentatricopeptide repeat (*PPR*) data, Roy and Lindqvist (2015) also recovered a close relationship among *Craniotome*, *Anisomeles*, and *Pogostemon* (*Microtoena* not sampled). In our analyses, however, *Craniotome* consistently grouped with *Paralamium* with high support values (Figures 2–4). Some morphological characters support the close relationship between *Paralamium* and *Craniotome*. For example, the size of pollen grains is very similar in *Paralamium* (16.3 × 15.0 μm) and *Craniotome* (16.6 × 14.9 μm), and are smaller than other lamioid genera (Abu-Asab and Cantino, 1994). Additionally, nutlet morphology supports the sister relationship between *Paralamium* and *Craniotome*. Nutlets in both genera are obovoid and glossy (Figures 7O,S) and have reticulate ornamentation on the surface (Figure 7P, × 750; Figure 7T, × 1200), while in other related genera in clade B (Figure 3), nutlets are oblong (*Leucosceptrum*, Figures 7A–C), hooked (*Rostrinucula*, Figures 6Q–S) or hairy (*Achyrospermum*, Figures 7I–J) at apex, or has eglaudular (*Comanthosphace*, Figure 6X) or glandular (*Eurysolen*, Figure 7G; *Colebrookea*, Figure 7X) trichomes. At the same time, *Paralamium* has some unique morphological characters, especially its unequal calyx lobs (i.e., 1/2/2 split), can differentiate it from other genera (calyx lobs 3/2 or 1/4 split, or (sub)equal) within Pogostemoneae.

### Circumscription and Relationships Within Pogostemoneae

The monophyly of Pogostemoneae was supported by most studies (Scheen et al., 2010; Bendiksby et al., 2011; Chen et al., 2014) based on cpDNA sequences, but not by Roy and Lindqvist (2015) using *PPR* data, who revealed that genera of Pogostemoneae were included in two separate clades. The first clade was referred as the *Achyrospermum* group (i.e., subclade A in Figure 2 sensu Roy and Lindqvist, 2015), forming the first-diverging clade within Lamioideae. The second clade consist of *Pogostemon*, *Anisomeles*, and *Craniotome* (i.e., subclade B in Figure 2 sensu Roy and Lindqvist, 2015), forming the second diverging clade sister to remainder of Lamioideae.

In our analyses, the cpDNA datasets strongly support the monophyly of Pogostemoneae (Figure 2), and the monophyly of this tribe was recovered based on nrITS dataset, although only two genera were selected as outgroup (Figure 3). Based on the results from present as well as previous studies (Scheen et al., 2010; Bendiksby et al., 2011; Chen et al., 2014; Roy and Lindqvist, 2015), Pogostemoneae comprises 12 genera: *Pogostemon*, *Anisomeles*, *Microtoena*, *Rostrinucula*, *Comanthosphace*, *Leucosceptrum*, *Eurysolen*, *Achyrospermum*, *Holocheila*, *Craniotome*, *Paralamium*, and *Colebrookea*. Most genera are monotypic or oligotypic, excepting *Pogostemon* (80 spp.), *Achyrospermum* (ca. 25 spp.), and *Microtoena* (19 spp.). Morphologically, Pogostemoneae is a very heterogeneous group, and synapomorphies for the tribe are still unclear. However, some morphological and anatomical characters can be used to distinguish Pogostemoneae from other lamioid members. Most genera of Pogostemoneae possess small and relatively glossy nutlets with pericarps often lacking a sclerenchyma region (Ryding, 1994a, 1995), generally long-exserted stamens with bearded filaments, weakly 2-lipped corollas, and broad bracts (Scheen et al., 2010). Additionally, pollen grains of Pogostemoneae are typically smaller (less than 28 × 27 μm) than that of most genera of Lamioideae (Abu-Asab and Cantino, 1994).

In addition to the confirmation of the systematic position of *Paralamium* and sister relationship between *Paralamium* and *Craniotome*, some other well supported groups within Pogostemoneae are also recovered in this study, which enables us to further discuss the relationships within the tribe. Based on nrITS phylogeny (Figure 3), two subclades (i.e., clade A and clade B) can be recognized. Clade A is strongly supported and composed of three genera (*Pogostemon*, *Anisomeles*, and *Microtoena*), while clade B is composed of the remaining genera of Pogostemoneae. Although clade B is weakly supported (0.59, -), this split is supported by nutlet morphology. In the present study, nutlets of 17 species representing 11 out of 12 genera (except *Holocheila*) of Pogostemoneae were included for analyses. Based on our LM and SEM observations, we found that nutlets of genera in clade A (Figure 3; *Pogostemon*, *Anisomeles*, and *Microtoena*) are glossy and relatively glabrous (Figures 5, 6A–P), and the sclerenchyma region is very distinctive (Bendiksby et al., 2011), while genera in clade B (*Rostrinucula*, *Comanthosphace*, *Leucosceptrum*, *Eurysolen*, *Achyrospermum*, *Paralamium*, *Craniotome*) have dull and glandular nutlets (Figures 7Q–X), and the sclerenchyma region is often absent or indistinct (Ryding, 1994a, 1995).

Within clade A, *Anisomeles* is sister to *Pogostemon*, with *Microtoena* sister to the *Anisomeles*-*Pogostemon* clade (Figures 3, 4). The three genera form a clade referred as clade A, which was supported by previous molecular phylogenetic studies (Scheen et al., 2010; Bendiksby et al., 2011). Cantino (1990, 1992a, 1992b)) suggested a close relationship between *Anisomeles* and *Pogostemon* based on their bearded staminal filaments and lustrous pericarps, as well as the presence of minute glands with unicellular caps on the leaf epidermis. Later, Abu-Asab and Cantino (1994) found that the two genera have very similar pollen grains with regular polygonal lumina and large perforations (see also Bean, 2015).

The close relationship between *Microtoena* and the *Pogostemon*-*Anisomeles* clade has been reported in previous studies (Scheen et al., 2010; Bendiksby et al., 2011; Chen et al., 2014; Roy and Lindqvist, 2015). The three genera are similar in terms of calyx morphology, with the calyx splitting the upper two and bottom three lobes up to ca. 1/2 of its length. Furthermore, linear bracts are present in *Anisomeles* and most species of *Microtoena*, while lanceolate or ovate bracts can be found in some species of *Microtoena* and *Pogostemon* (Wang, 2018). Geographically, most of the species of clade A are distributed in tropical East Asia (Scheen et al., 2010), although some species occur on islands within the Pacific and West Indian Oceans (*Anisomeles*), Africa (*Pogostemon*), and the Himalayas (*Craniotome* and *Pogostemon glaber*).

*Microtoena* was shown to be polyphyletic in some studies (Bendiksby et al., 2011; Roy and Lindqvist, 2015) based on cpDNA regions, but our results recover it as monophyletic with convincing support (Figure 3). A possible reason for this discrepancy may be that only two species and three cpDNA markers (*mat*K, *trn*L-*trn*F, *rps*16 intron) were used in previous studies. Wang (2018) included 11 species for the phylogenetic reconstruction of *Microtoena*. Though his study was based only on two cpDNA regions (*mat*K, *trn*L-*trn*F), the monophyly of *Microtoena* was well supported, as in our present study using nrITS (Figure 3, 100%, 1.00) and additional cpDNA markers (Figure 4, 74%, 0.98). *Microtoena* is a poorly understood genus and was previously placed within Stachydeae (Prain, 1889; Briquet (1895–1897)). Although recent molecular phylogenetic studies (Bendiksby et al., 2011; Yao et al., 2016; Zhao et al., 2021) confirmed its placement within Pogostemoneae, corroborating the taxonomic treatment of Harley et al. (2004), species relationships within *Microtoena* remain unresolved.

Another subclade (i.e., *Achyrospermum* group) composed of *Achyrospermum*, *Eurysolen*, *Leucosceptrum*, *Rostrinucula*, and *Comanthosphace* is also strongly supported in both the nrITS (Figure 3) and cpDNA trees (Figure 4), among which *Rostrinucula* and *Comanthosphace* are consistently resolved as sister genera (Figures 3, 4). The *Achyrospermum* group was first reported by Bendiksby et al. (2011) using cpDNA markers and subsequently recovered by Roy and Lindqvist (2015) based on the *PPR* region, but neither of them sampled *Leucosceptrum*. Species of the *Achyrospermum* group are distributed mainly in tropical East Asia and share several morphological characters. For example, the sclerenchyma region in the fruit pericarp is present in most lamioid members (Ryding, 1995; Bendiksby et al., 2011), but is obsolete, indistinct, or absent in the *Achyrospermum* group (Ryding, 1994b, 1995). Moreover, genera in this subclade have dull and glandular nutlets (Figures 6Q–X, 7A–L), while other genera within Pogostemoneae have glossy and glabrous nutlets (Bendiksby et al., 2011). Stamens long-exserted from the corolla are rare in Lamioideae, and are restricted to *Comanthosphace*, *Rostrinucula*, and *Leucosceptrum* in the *Achyrospermum* group, as well as a few species of *Pogostemon* in clade A. As suggested by Scheen et al. (2010), this character may be a synapomorphy for the small clade consisting of *Comanthosphace*, *Rostrinucula*, and *Leucosceptrum*. Molecular phylogenetic and morphological studies based on a broader sampling and more DNA sequences may further help to elucidate relationships within Pogostemoneae and identify morphological synapomorphies for the tribe.

### Incongruence Between Nuclear and Plastid Phylogenies

In this study we provide the first comprehensive molecular phylogenetic study of Pogostemoneae. Though the intergeneric relationships within this tribe are generally well resolved, the placement of four monotypic genera (*Colebrookea*, *Holocheila*, *Paralamium* and *Craniotome*) is still uncertain due to incongruent topologies between nrITS and cpDNA trees. In the nrITS phylogeny (Figure 3), the *Paralamium*-*Craniotome* clade is sister to *Colebrookea* but weakly supported (57%, -). The *Paralamium*-*Craniotome*-*Colebrookea* clade is then sister to a clade including *Holocheila* and the *Achyrospermum* group, which is also weakly supported (-, 0.90) again. In the cpDNA tree (Figure 4), however, *Holocheila* is the first diverging clade, followed by *Colebrookea*, the *Achyrospermum* group, and then *Paralamium*-*Craniotome _+_* clade A, which is largely consistent with the topology of Chen et al. (2014). Most genera in clade B (excepting *Achyrospermum*, 25 spp.), all other genera are monotypic (*Colebrookea*, *Craniotome, Eurysolen*, *Holocheila*, *Leucosceptrum, Paralamium, Rostrinucula*) or oligotypic (*Comanthosphace*, 4 spp.) and mainly distributed in East Asia.

Incongruence between genomes have been noted within several genera in Lamiaceae, and ancient hybridization and chloroplast capture has often been posited to have contributed to the discordance (e.g., Albaladejo et al., 2005; Drew and Sytsma, 2013; Drew et al., 2014; Deng et al., 2015; Walker et al., 2015; Hu et al., 2018). Roy and Lindqvist (2015) suggested ancient reticulation events are likely to be responsible for the discordance between the plastid and *PPR* topologies of Pogostemoneae. They also demonstrated that ancestors of Pogostemoneae may have undergone rapid diversification during the middle Miocene in East Asia, which may have been triggered by climatic changes resulting from the uplift of the Qinghai-Tibetan Plateau (QTP) (Roy and Lindqvist, 2015). Considering that incomplete lineage sorting (ILS) among taxa is often associated with rapid radiations (Enard and Paabo, 2004; Pollard et al., 2006), ILS may also be a cause of the incongruences between the nuclear and plastid trees of Pogostemoneae. In the present study, two clades (clade A and clade B) are recognized based on nrITS phylogeny, but clade B is weakly supported by nrITS data and not recovered using cpDNA data. Although nutlet morphology supported the division of these two clades, futures studies involving next-generation sequencing and increased taxon sampling are need to provide insights into the complex evolutionary history of this group.

### Key to All Genera of Pogostemoneae

The following circumscription of Pogostemoneae is based on this as well as previous studies (Scheen et al., 2010; Bendiksby et al., 2011). We provide a key to the 12 genera of Pogostemoneae below.

1 Creeping herb; corolla with two entire lips (1/1)…………………………………… ***Holocheila***
1 Shrub, subshrubs or erect herb; corolla 2-lipped, 4-lobed (1/3, 1/3 or 2/3)…………………………………… 2
2 Calyx 5-lobed, lobes unequal (1/2/2), posterior lip very broad ………………………………… ***Paralamium***
2 Calyx 5-lobed unequal (3/2 or 1/4) or (sub)equal …… 3
3 Flowers dioecious with dimorphic male and female flowers ………………………………………… ***Colebrookea***
3 Flowers monoecious ……………………………… 4
4 Corolla-tube longer than 1.5 cm …………… ***Microtoena***
4 Corolla-tube less than 1 cm long …………………… 5
5 Nutlet narrowly ellipsoid, hooked at apex … ***Rostrinucula***
5 Nutlet not narrowly ellipsoid, unhooked at apex ……… 6
6 Filaments usually bearded along center with moniliform hairs …………………………………… ***Pogostemon***
6 Filaments without moniliform hairs ………………… 7
7 Stamens long-exserted from corolla ………………… 8
7 Stamens not or shortly exserted from corolla ………… 9
8 Shrub or small tree; nutlets cylindrical-oblong ……………………………………… ***Leucosceptrum***
8 Rhizomatous perennial herbs; nutlets obovate ……………………………………***Comanthosphace***
9 Nutlets scaly at apex ………………… ***Achyrospermum***
9 Nutlets never scaly at apex ………………………… 10
10 Anthers 1-celled; corolla tube saccate in front … ***Eurysolen***
10 Anthers 2-celled; corolla tube not saccate in front …… 11
11 Verticillasters in dense or interrupted, long, terminal spikes ………………………………………… ***Anisomeles***
11 Cymes pedunculate, helicoid or sometimes dichotomous, in axillary or terminal panicles ………………………………… ***Craniotome***

## Conclusion

This study confirms the systematic placement of *Paralamium* for the first time inferred from chloroplast and nuclear DNA data. *Paralamim* is a member of the tribe Pogostemoneae within Lamioideae and is sister to *Craniotome*. As currently defined, the tribe Pogostemoneae is composed of 12 genera, and the monophyly of Pogostemoneae is supported in all analyses. Phylogenetically, Pogostemoneae are the first diverging tribe and sister to the remaining Lamioideae. Morphologically, Pogostemoneae are a remarkably diverse group and lack clear synapomorphies. Although some well-supported groups were identified within Pogostemoneae, relationships of some monotypic genera (e.g., *Holocheila*, *Colebrookea*, *Paralamium* and *Craniotome*) remain unclear. Thus, studies using broad sampling of low-copy and/or single-copy intrageneric phylogenies and detailed comparative morphological investigation are needed.

## Data Availability Statement

The datasets presented in this study can be found in online repositories. The names of the repository/repositories and accession number(s) can be found in the article/Supplementary Material.

## Author Contributions

FZ, C-LX, BD, and BL conceived the idea and designed the research. Y-WW, FZ, and Y-PC conducted experiments. E-DL and JC conducted specimen and seed collection. FZ, Y-WW, Y-PC, GY, BD, JC, E-DL, BL, and C-LX wrote the manuscript. All authors contributed to the article and approved the submitted version.

## Conflict of Interest

The authors declare that the research was conducted in the absence of any commercial or financial relationships that could be construed as a potential conflict of interest.

## Footnotes

1 https://code.google.com/p/ea-utils/

2 http://www.Phylo.org

## Supplementary Material

Supplementary Figure 1 Gene map of the complete chloroplast genome of *Paralamium griffithii*. Genes inside and outside of the circle are transcribed in the clockwise and counterclockwise directions, respectively. Genes belonging to different functional categories are color-coded.

Supplementary Figure 2 Maximum likelihood phylogeny of Lamiaceae based on 79 chloroplast DNA regions (dataset CP79) of coding regions dataset, ambiguous sites were excluded for analyses. Maximum likelihood bootstrap support (MLBS) are near the branches. A “–” indicates MLBS values < 50%.

Supplementary Figure 3 Phylograms from Bayesian Inference (BI) analyses of Lamiaceae based on concatenated nucleotide sequences of 79 protein-coding genes (dataset CP79), ambiguous sites were excluded for analyses. Bayesian inference posterior probability (BIPP) are near the branches. A “–” indicates BIPP values < 0.8.

Supplementary Figure 4 Maximum likelihood phylogeny of Pogostemoneae based on the nrITS region, ambiguous sites were excluded for analyses. Maximum likelihood bootstrap support (MLBS) are near the branches. A “–” indicates MLBS values < 50%.

Supplementary Figure 5 Phylograms from Bayesian Inference (BI) analyses of Pogostemoneae based on the nrITS region, ambiguous sites were excluded for analyses. Bayesian inference posterior probability (BIPP) are near the branches. A “–” indicates BIPP values < 0.8.

Supplementary Figure 6 Maximum likelihood phylogeny of Pogostemoneae based on the combined dataset of five cpDNA regions (*mat*K, *rbc*L, *rps*16, *trn*H-*psb*A, and *trn*L-*trn*F), ambiguously aligned sites were excluded from analyses. Maximum likelihood bootstrap support (MLBS) are near the branches. A “–” indicates MLBS values < 50%.

Supplementary Figure 7 Phylograms from Bayesian Inference (BI) analyses of Pogostemoneae based on the combined dataset of five cpDNA regions(*mat*K, *rbc*L, *rps*16, *trn*H-*psb*A, and *trn*L-*trn*F), ambiguously aligned sites were excluded from analyses. Bayesian inference posterior probability (BIPP) are near the branches. A “–” indicates BIPP values < 0.8.

Supplementary Figure 8 Phylograms from Bayesian Inference (BI) analyses of Pogostemoneae based on the combined dataset of five cpDNA regions (*mat*K, *rbc*L, *rps*16, *trn*H-*psb*A, and *trn*L-*trn*F), under a partitioned strategy model, ambiguously aligned sites were excluded from analyses. Bayesian inference posterior probability (BIPP) are near the branches. A “–” indicates BIPP values < 0.8.

Supplementary Table 1 List of taxa sampled with information related to taxonomy, GenBank accession numbers and references.

Supplementary Table 2 Features of newly sequenced plastome of *Paralamium griffithii*.

Supplementary Table 3 Excluded ambiguous sites for each dataset.
