# Supplementary figures and images for: Systematic Placement of the Enigmatic Southeast Asian Genus Paralamium and an Updated Phylogeny of Tribe Pogostemoneae (Lamiaceae Subfamily Lamioideae)
Source: Front Plant Sci. 2021 Apr 16;12:646133. doi: 10.3389/fpls.2021.646133 (PMC8085563; doi:10.3389/fpls.2021.646133)

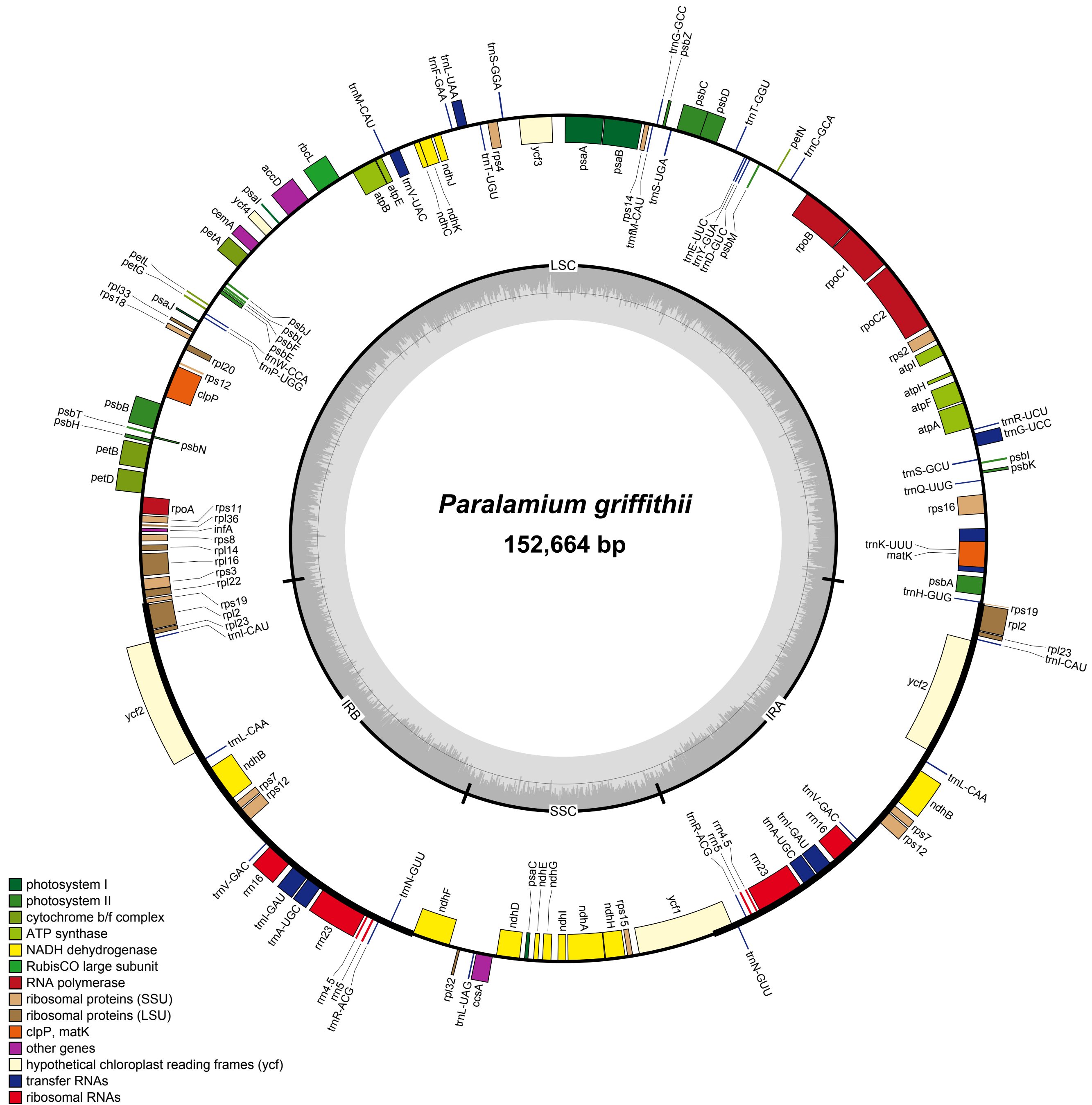

Supplement: Supplementary Figure 1 — Gene map of the complete chloroplast genome of Paralamium griffithii. Genes inside and outside of the circle are transcribed in the clockwise and counterclockwise directions, respectively. Genes belonging to different functional categories are color-coded. [file Image_1.pdf]

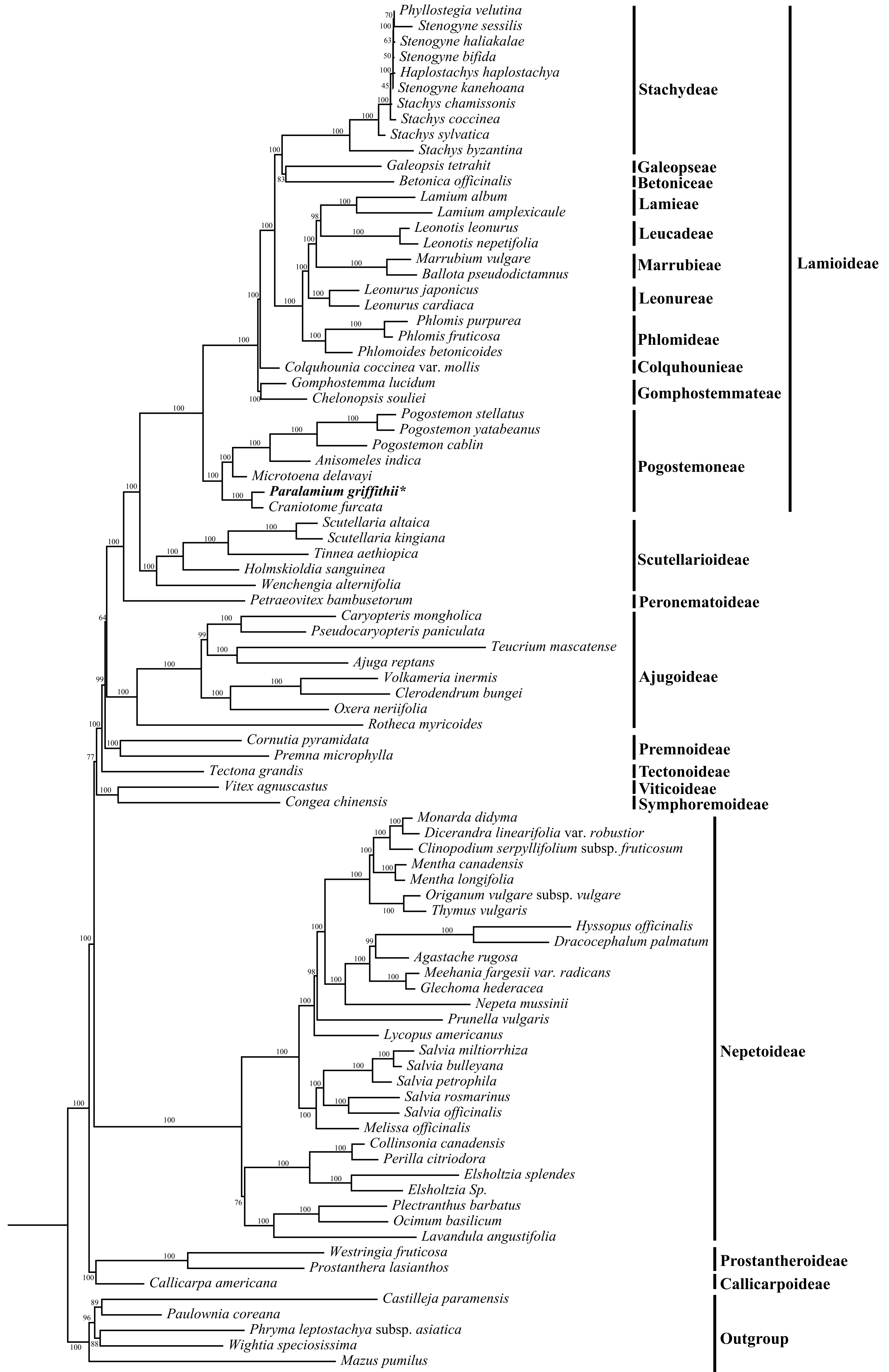

Supplement: Supplementary Figure 2 — Maximum likelihood phylogeny of Lamiaceae based on 79 chloroplast DNA regions (dataset CP79) of coding regions dataset, ambiguous sites were excluded for analyses. Maximum likelihood bootstrap support (MLBS) are near the branches. A “–” indicates MLBS values < 50%. [file Image_2.pdf]

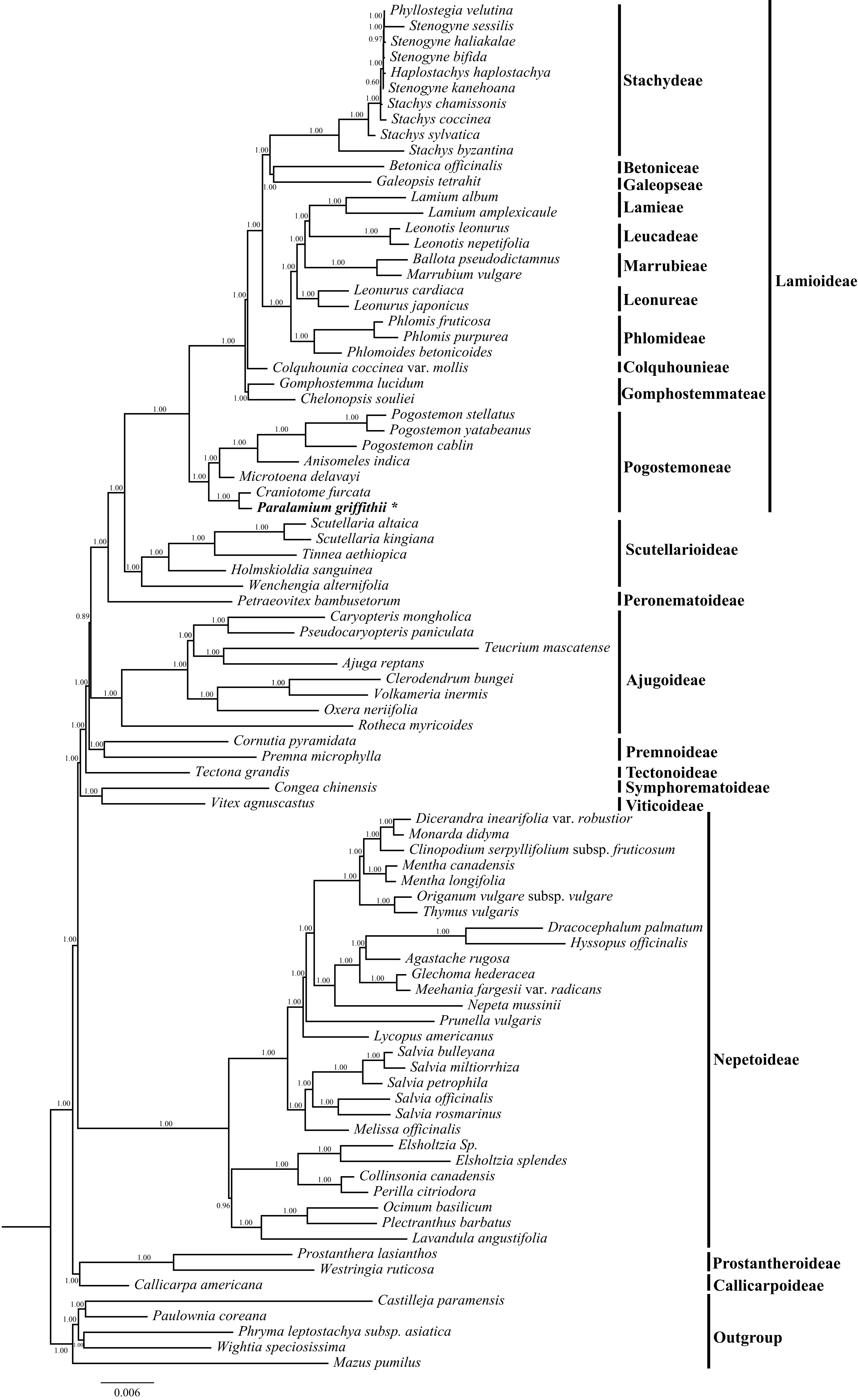

Supplement: Supplementary Figure 3 — Phylograms from Bayesian Inference (BI) analyses of Lamiaceae based on concatenated nucleotide sequences of 79 protein-coding genes (dataset CP79), ambiguous sites were excluded for analyses. Bayesian inference posterior probability (BIPP) are near the branches. A “–” indicates BIPP values < 0.8. [file Image_3.pdf]

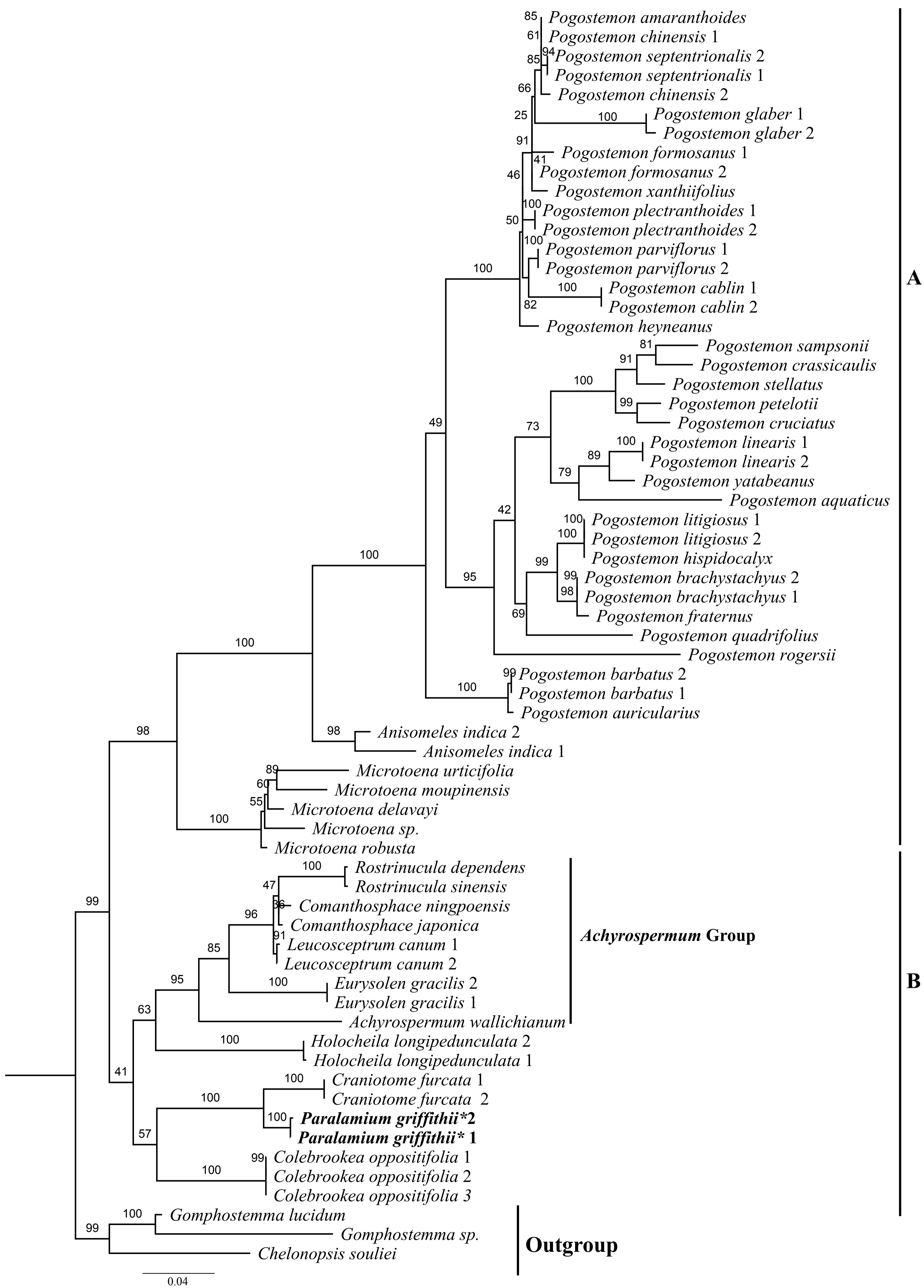

Supplement: Supplementary Figure 4 — Maximum likelihood phylogeny of Pogostemoneae based on the nrITS region, ambiguous sites were excluded for analyses. Maximum likelihood bootstrap support (MLBS) are near the branches. A “–” indicates MLBS values < 50%. [file Image_4.pdf]

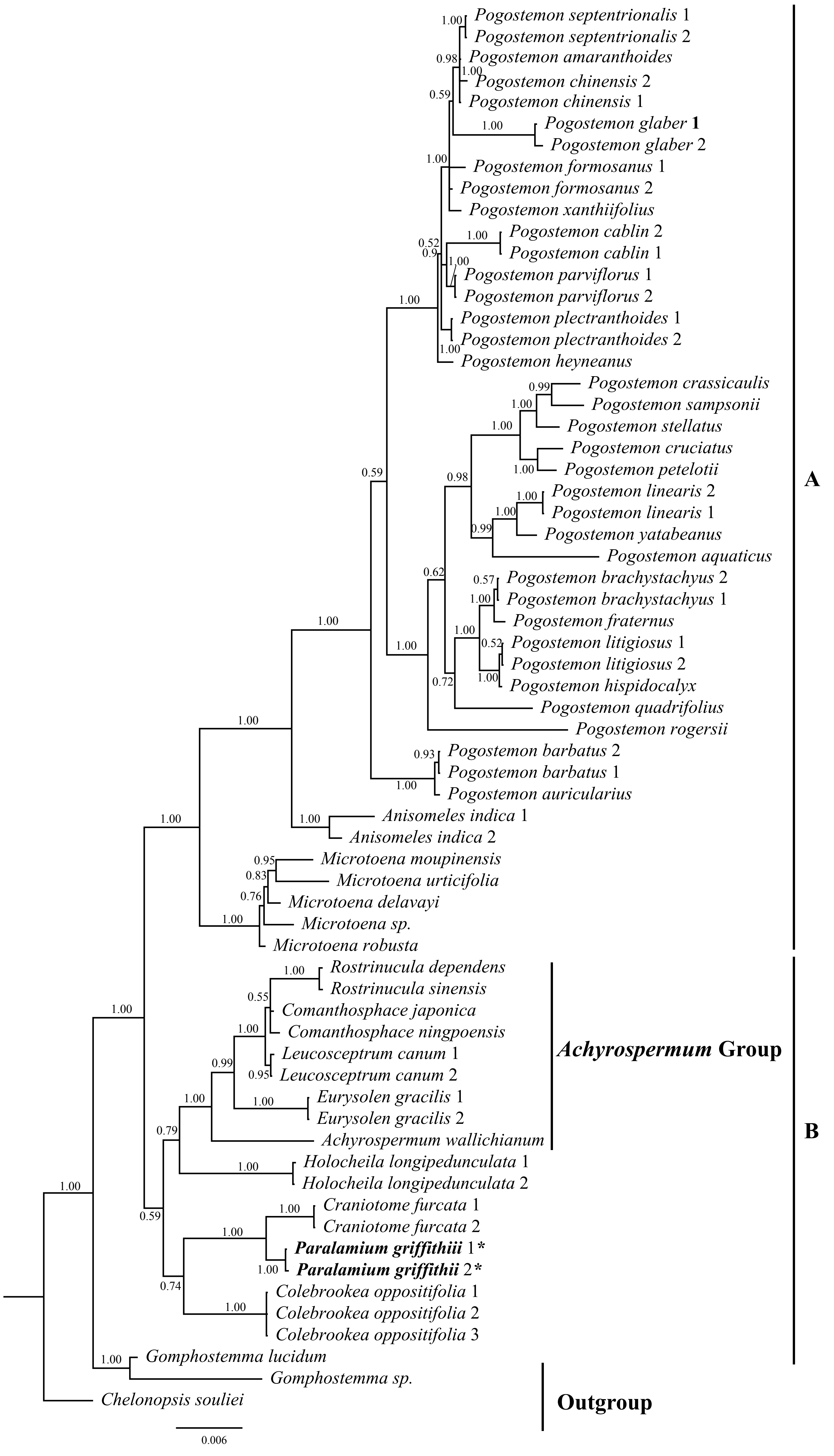

Supplement: Supplementary Figure 5 — Phylograms from Bayesian Inference (BI) analyses of Pogostemoneae based on the nrITS region, ambiguous sites were excluded for analyses. Bayesian inference posterior probability (BIPP) are near the branches. A “–” indicates BIPP values < 0.8. [file Image_5.pdf]

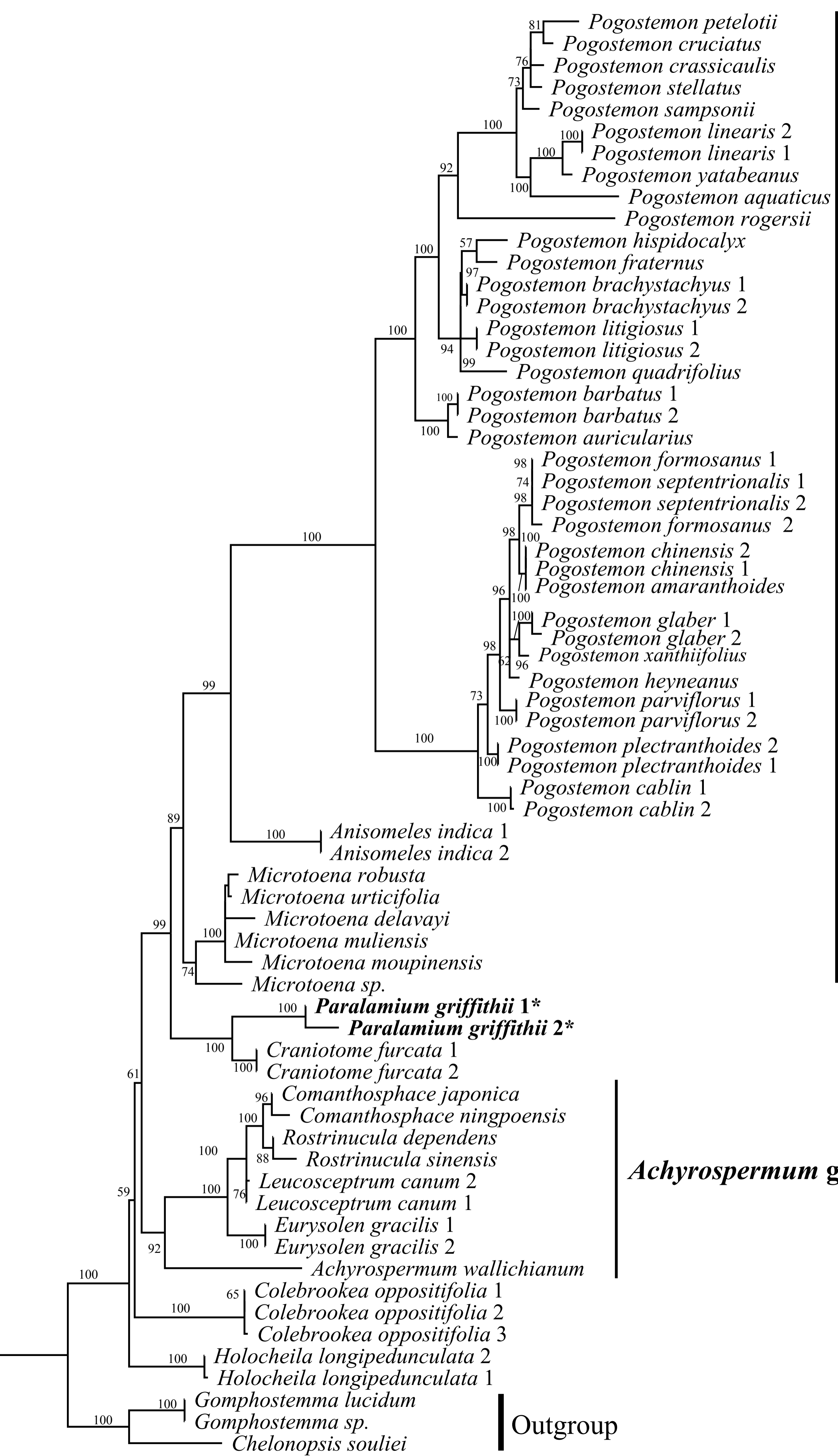

A

*Achyrospermum* group

Outgroup

0.005

Supplement: Supplementary Figure 6 — Maximum likelihood phylogeny of Pogostemoneae based on the combined dataset of five cpDNA regions (matK, rbcL, rps16, trnH-psbA, and trnL-trnF), ambiguously aligned sites were excluded from analyses. Maximum likelihood bootstrap support (MLBS) are near the branches. A “–” indicates MLBS values < 50%. [file Image_6.pdf]

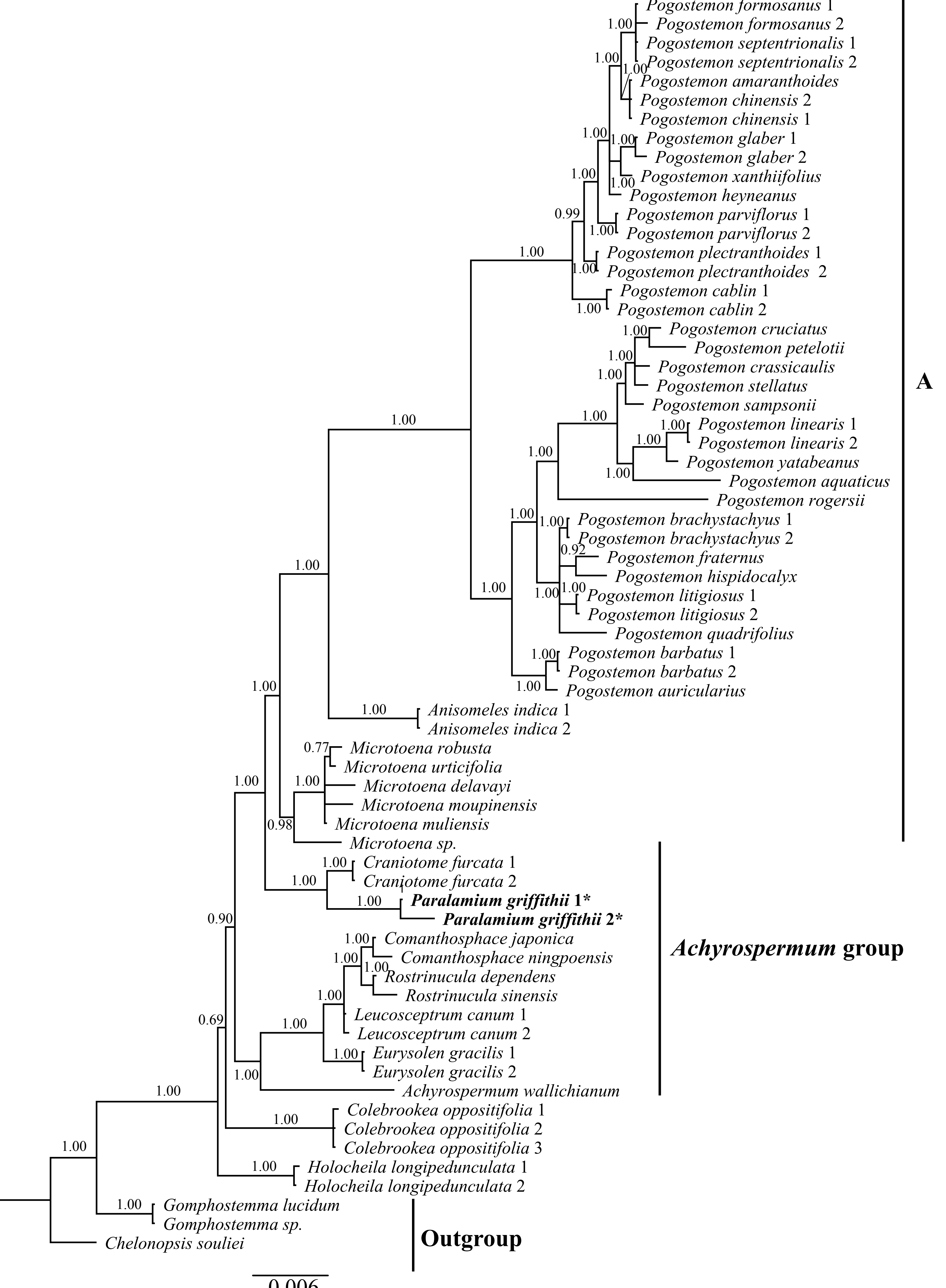

Supplement: Supplementary Figure 7 — Phylograms from Bayesian Inference (BI) analyses of Pogostemoneae based on the combined dataset of five cpDNA regions(matK, rbcL, rps16, trnH-psbA, and trnL-trnF), ambiguously aligned sites were excluded from analyses. Bayesian inference posterior probability (BIPP) are near the branches. A “–” indicates BIPP values < 0.8. [file Image_7.pdf]

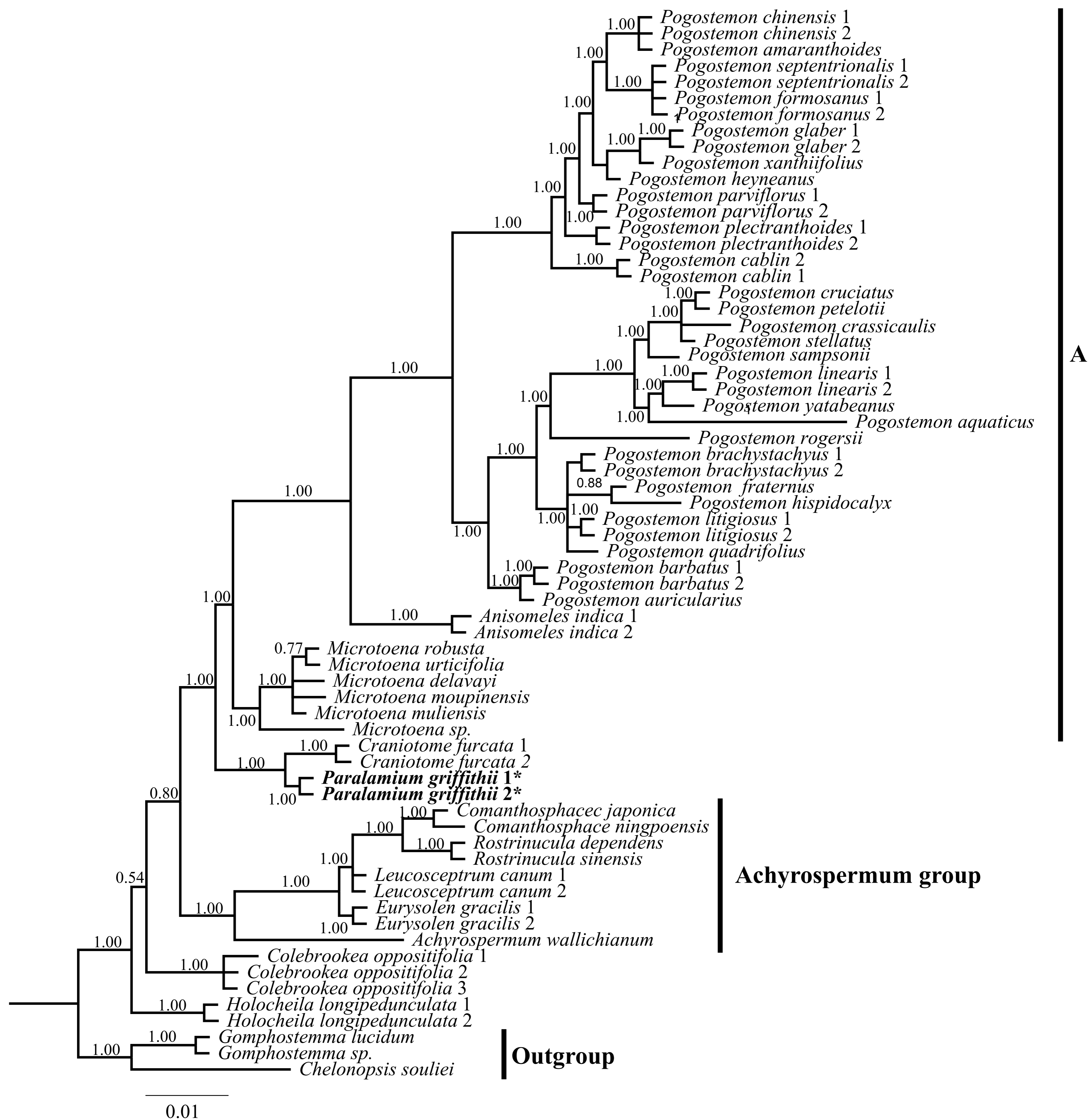

Supplement: Supplementary Figure 8 — Phylograms from Bayesian Inference (BI) analyses of Pogostemoneae based on the combined dataset of five cpDNA regions (matK, rbcL, rps16, trnH-psbA, and trnL-trnF), under a partitioned strategy model, ambiguously aligned sites were excluded from analyses. Bayesian inference posterior probability (BIPP) are near the branches. A “–” indicates BIPP values < 0.8. [file Image_8.pdf]
