# Supplementary material for: Systematic Placement of the Enigmatic Southeast Asian Genus Paralamium and an Updated Phylogeny of Tribe Pogostemoneae (Lamiaceae Subfamily Lamioideae)
Source: Front Plant Sci. 2021 Apr 16;12:646133. doi: 10.3389/fpls.2021.646133 (PMC8085563; doi:10.3389/fpls.2021.646133)
Supplement: Supplementary Table 2 — Features of newly sequenced plastome of Paralamium griffithii. [file Table_2.DOCX]

**Table S2 The gene functions of the plastome of *Paralamium griffithii.***

| **Category for genes** | **Group of genes** | **Name of genes** |
| --- | --- | --- |
| Photosynthesis | Subunits of NADH-dehydrogenase | *ndhA*, ndhB**(2x), *ndhC, ndhD, ndhE, ndhF, ndhG, ndhH, ndhI, ndhJ, ndhK* |
|  | Photosystem I | *psaA, psaB, psaC, psaI, psaJ, ycf3*** |
|  | Photosystem II | *psbA, psbB, psbC, psbD, psbE, psbF, psbH, psbI, psbJ, psbK, psbL, psbM, psbN, psbT, psbZ* |
|  | Cytochrome b/f complex | *petA, petB*, petD*, petG, petL, petN* |
|  | ATP synthase | *atpA, atpB, atpE, atpF*, atpH, atpI* |
|  | Large chain of rubisco | *rbcL* |
| Self-replication | Ribosomal RNA genes | *rrn16* (2x), *rrn23* (2x), *rrn4.5* (2x), *rrn5* (2x) |
|  | Transfer RNA genes 30 tRNA genes | (6 contain one intron, 7 are duplicated in the IR region) |
|  |  | *trnA-UGC**(2x)*, trnfM-CAU, trnI-GAU**(2x)*, trnM-CAU, trnR-ACG*(2x), *trnS-UGA, trnC-GCA, trnG-GCC*, trnK-UUU*, trnN-GUU(2x), trnW-CCA, trnT-GGU, trnD-GUC, trnG-UCC, trnL-CAA(2x), trnY-GUA,*  *trnR-UCU, trnT-UGU, trnE-UUC, trnH-GUG, trnL-UAA*, trnP-UGG, trnS-GCU, trnV-GAC(2x), trnF-GAA, trnI-CAU(2x), trnL-UAG, trnQ-UUG, trnS-GGA, trnV-UAC** |
|  | Small subunit of ribosome | *rps2, rps3, rps4, rps7* (2x)*, rps8, rps11, rps12, rps14, rps15, rps16*, rps18, rps19* |
|  | Large subunit of ribosome | *rpl2** (2x)*, rpl14, rpl16*, rpl20, rpl22, rpl23* (2x)*, rpl32, rpl33, rpl36* |
|  | RNA polymerase subunits | *rpoA, rpoB, rpoC1*, rpoC2* |
| Other genes | Translation initiation factor | *infA* |
|  | Maturase | *matK* |
|  | Protease | *clpP*** |
|  | Envelope membrane protein | *cemA* |
|  | Subunit of acetyl-CoA-carboxylase | *accD* |
|  | cytochrome c biogenesis protein | *ccsA* |
|  | Component of TIC complex | *ycf1* |
| Genes of unknown function | | *ycf2, ycf4, ycf15* (2x) |

Notes: *gene with a single intron, **gene with two introns, (2x) duplicated gene.
